# 5′-Guanidino Xylofuranosyl Nucleosides as Novel Types of 5′-Functionalized Nucleosides with Biological Potential

**DOI:** 10.3390/ph18050734

**Published:** 2025-05-16

**Authors:** Jennifer Szilagyi, Tânia Moreira, Rafael Santana Nunes, Joana Silva, Celso Alves, Alice Martins, Rebeca Alvariño, Niels V. Heise, René Csuk, Nuno M. Xavier

**Affiliations:** 1 Centro de Química Estrutural, Institute of Molecular Sciences, Faculdade de Ciências, Universidade de Lisboa, Ed. C8, 5° Piso, Campo Grande, 1749-016 Lisboa, Portugal; edinajennifer@gmail.com (J.S.); tmmoreira@fc.ul.pt (T.M.); rsnunes@fc.ul.pt (R.S.N.); 2 BioISI—Instituto de Biosistemas e Ciências Integrativas, Faculdade de Ciências, Universidade de Lisboa, 1749-016 Lisboa, Portugal; 3 MARE—Marine and Environmental Sciences Centre & ARNET—Aquatic Research Network Associated Laboratory, ESTM, Polytechnic University of Leiria, Av. Porto de Pesca, Edifício Cetemares, 1749-016 Lisboa, Portugal; joana.m.silva@ipleiria.pt (J.S.); celso.alves@ipleiria.pt (C.A.); alice.martins@ipleiria.pt (A.M.); 4 Departamento de Fisiología, Facultade de Veterinaria, IDIS, Universidade de Santiago de Compostela, 27002 Lugo, Spain; rebeca.alvarino@usc.es; 5 Bereich Organische Chemie, Martin-Luther-Universität Halle-Wittenberg, Kurt-Mothes-Str. 2, D-06120 Halle (Saale), Germany; niels.heise@chemie.uni-halle.de (N.V.H.); rene.csuk@chemie.uni-halle.de (R.C.); 6 Research Institute for Medicines (iMed.ULisboa), Faculty of Pharmacy, Universidade de Lisboa, Av. Prof. Gama Pinto, 1649-003 Lisboa, Portugal

**Keywords:** nucleos(t)ide analogs, anticancer agents, cholinesterase inhibitors, neuroprotective compounds, guanidine moiety, N-glycosylatiom

## Abstract

**Background/Objectives:** While various nucleoside and nucleotide analogs have been approved as anticancer and antiviral drugs, their limitations, including low bioavailability and chemotherapeutic resistance, encourage the development of novel structures. In this context, and motivated by our previous findings on bioactive 3′-*O*-substituted xylofuranosyl nucleosides and 5-guanidine xylofuranose derivatives, we present herein the synthesis and biological evaluation of 5′-guanidino furanosyl nucleosides comprising 6-chloropurine and uracil moieties and a 3-*O*-benzyl xylofuranosyl unit. **Methods:** The synthetic methodology was based on the *N-*glycosylation of a 5-azido 3-*O*-benzyl xylofuranosyl acetate donor with the silylated nucleobase and a subsequent one-pot sequential two-step protocol involving Staudinger reduction of the thus-obtained 5-azido uracil and N^7^/N^9^-linked purine nucleosides followed by guanidinylation with *N*,*N*′-bis(*tert*-butoxycarbonyl)-*N*′′-triflylguanidine. The molecules were evaluated for their anticancer and anti-neurodegenerative diseases potential. **Results:** 5′-Guanidino 6-chloropurine nucleosides revealed dual anticancer and butyrylcholinesterase (BChE)-inhibitory effects. Both N^9^/N^7^-linked nucleosides exhibited mixed-type and selective submicromolar/micromolar BChE inhibiton. The N^9^ regioisomer was the best inhibitor (*K*_i_/*K*_i_′ = 0.89 μM/2.96 μM), while showing low cytotoxicity to FL83B hepatocytes and no cytotoxicity to human neuroblastoma cells (SH-SY5Y). Moreover, the N^9^-linked nucleoside exhibited selective cytotoxicity to prostate cancer cells (DU-145; IC_50_ = 27.63 μM), while its N^7^ regioisomer was active against all cancer cells tested [DU-145, IC_50_ = 24.48 μM; colorectal adenocarcinoma (HCT-15, IC_50_ = 64.07 μM); and breast adenocarcinoma (MCF-7, IC_50_ = 43.67 μM)]. In turn, the 5′-guanidino uracil nucleoside displayed selective cytotoxicity to HCT-15 cells (IC_50_ = 76.02 μM) and also showed neuroprotective potential in a Parkinson’s disease SH-SY5Y cells’ damage model. The active molecules exhibited IC_50_ values close to or lower than those of standard drugs, and comparable, or not significant, neuro- and hepatotoxicity. **Conclusions**: These findings demonstrate the interest of combining guanidine moieties with nucleoside frameworks towards the search for new therapeutic agents.

## 1. Introduction

**Figure 1:**
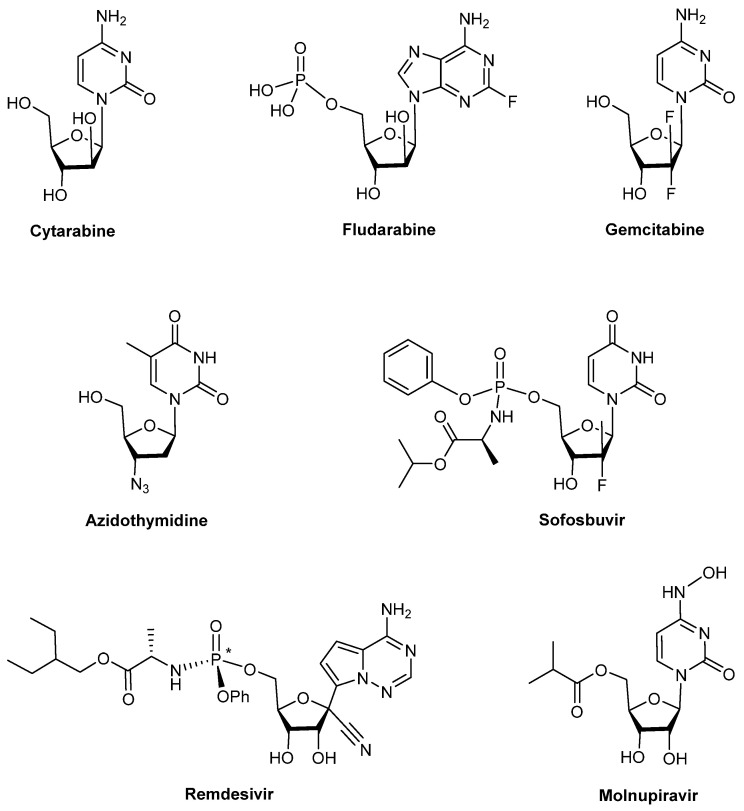
Representative examples of clinically used nucleos(t)ide analogues against cancer (cytarabine, fludarabine, and gemcitabine) and viral infections (azidothymidine, sofosbuvir, remdesivir, and molnupiravir).

The synthesis of nucleoside and nucleotide analogs and mimetics has occupied a prominent place in organic chemistry, particularly in the context of drug discovery, due to their ability to exhibit a variety of biological activities. Such compounds have the propensity to mimic their physiological counterparts and interfere with their roles in essential biological processes, such as nucleic acid synthesis, nucleotide metabolism, or cell signaling, which are crucial for life but also important for the development and progression of diseases, such as cancer or viral infections [1,2,3]. In this context, several molecules from these groups became approved effective anticancer and antiviral drugs, acting mostly as nucleic acid antimetabolites. Among the clinically used nucleos(t)ide analogs are the anticancer drugs cytarabine [4], fludarabine [5], gemcitabine [6], or the antivirals azidothymidine [7], sofosbuvir [8] and the anti-COVID-19 agents remdesivir [9] and molnupiravir [10] (Figure 1). Other types of biological effects have been reported for nucleos(t)ide analogs. Some anticancer nucleoside antimetabolites also exhibit antibacterial activity by inhibiting DNA replication or nucleotide metabolism [11], while various nucleos(t)ide analogs have showed antibacterial effects as inhibitors of other important microbial cell processes, such as protein or cell-wall biosynthesis [12,13], although none of these molecules have become approved antibiotics [14]. Some reports have shown the interest in nucleoside analogs as potential anti-Alzheimer’s agents, with an ability to inhibit cholinesterases [15,16,17,18,19,20].

Despite their efficacy, the clinically used nucleos(t)ide analogs suffer from some drawbacks, including low oral bioavailability, unwanted toxicity, and the resistance of cancer or virus-infected cells towards their action [21]. Therefore, the development of novel nucleos(t)ide analogs possessing structures that allow them to overcome such limitations, displaying better abilities to penetrate cells and alternative mechanisms of action, is highly relevant. The search for new therapeutic applications and the exploitation of less studied biological properties of nucleoside analogues is also encouraging, given their propensity to exhibit bioactivity.

Modifications to the parent structures of nucleos(t)ides towards new potentially bioactive analogs include the use of other glycosyl moieties besides ribose or 2-deoxyribose, substituted purine or uracil derivatives or other nitrogeneous heteroaromatic systems, and the inclusion of phosphate group surrogates or mimetic groups.

**Figure 2:**
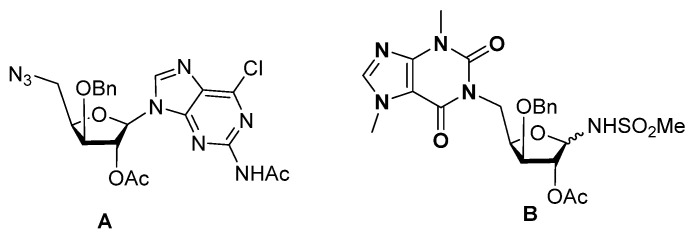
Bioactive nucleoside analogs comprising a 3-*O*-benzyl xylofuranosyl unit.

Our group has reported the synthesis and the significant biological profile of various types of nucleoside analogs based on a 3′-*O*-substituted xylofuranosyl unit. Among them are 5′-azido 3′-*O*-benzyl/dodecyl nucleosides, which showed significant antiproliferative effects in K562 leukemia cells and in breast cancer cells [22,23], with higher cell selectivity than their glucopyranosyl counterparts. In particular, 3-*O*-benzyl derivatives comprising a 2-acetamido-6-chloropurine motif were selectively active against K562 leukemia cells and were shown to act by inducing G2/M cell cycle arrest (**A**, Figure 2) [22]. Another example of a bioactive nucleoside analogue containing a 3-*O*-benzyl xylofuranosyl moiety is a theobromine isonucleosidyl sulfonamide (**B**, Figure 2), which exhibited selective and micromolar inhibition of acetylcholinesterase (AChE) [17]. Moreover, it did not show toxicity to normal fibroblasts or to a neuronal cell. AChE remains a major therapeutic target for Alzheimer’s disease symptomatic treatment, since it hydrolyses the neurotransmitter acetylcholine (ACh) in cholinergic synapses [24]. Moreover, it has been shown that AChE is also involved in the regulation of cell growth, survival, differentiation, proliferation, and resistance to apoptosis, which indicates it may play a role in cancer progress [25,26,27,28].

**Figure 3:**
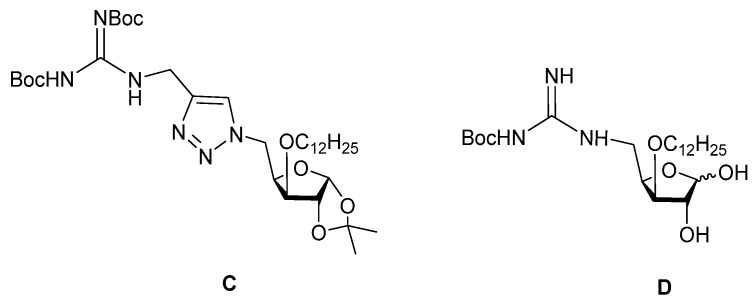
Bioactive guanidine-containing isonucleoside analogs comprising a xylofuranosyl unit.

3-*O*-Substituted xylofuranosyl units were also used as templates for isonucleoside analogs containing a terminal guanidine group, namely, 5-guanidino xylofuranose derivatives and a guanidinomethyltriazole 5′-isonucleoside (Figure 3) [29]. The latter compound (**C**) was shown to display moderate inhibition of AChE, while a 3-*O*-dodecyl 5-(*N*-Boc)guanidino xylofuranose derivative (**D**) was identified as a selective micromolar inhibitor of AChE. Moreover, the guanidine derivative **D** showed significant antiproliferative activities in K562 leukemia cells and in MCF-7 breast cancer cells, accompanied by a relatively low effect on normal fibroblasts.

Encouraged by our previous findings concerning the significant biological profile encountered for newly synthesized 3-*O*-benzyl xylofuranosyl nucleoside analogs, and for 5-guanidino xylofuranose derivatives, we report, herein, the synthesis of 5′-guanidino nucleosides comprising a 3-*O*-benzyl xylofuranosyl moiety and the evaluation of their anticancer and anti-neurodegenerative diseases potential. While the combination of guanidine moieties to nucleoside systems has been previously reported in the context of the synthesis of oligonucleotide mimetics in which the phosphodiester bond is replaced by a guanidine group [30], to the best of our knowledge, there are no reports on guanidino nucleosides. In such structures, a nucleoside is linked to another system that is a recognized pharmacophore moiety present in several bioactive compounds, having the ability for enzyme or receptor recognition through hydrogen bond or cation-π interactions [31,32,33]. In this study, 6-chloropurine was used as a purine derivative rather than 2-acetamido-6-chloropurine, which is contained in the previously reported anticancer 3-*O*-benzyl xylofuranosyl nucleosides. This modification was made to assess the aptitude of the 6-chloropurine moiety to confer bioactivity to the nucleosides.

## 2. Results and Discussion

### 2.1. Chemistry

**Scheme 1:**
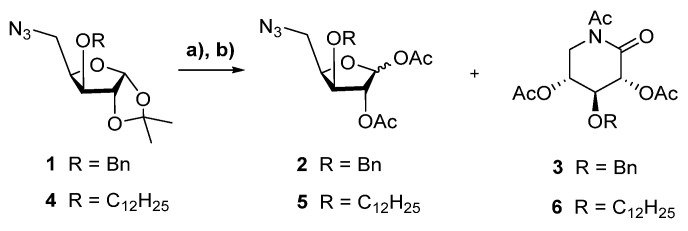
**Reagents and Conditions:** for **R = Bn**: (a) TFA (65% aq.), r.t., 30 min; (b) Ac_2_O, py, 1 h, to give only **2**, 80%, α/β = 1:1, 2 steps or (a) TFA (65% aq.), r.t., 2 h; (b) Ac_2_O, py, 1 h, **2** (α/β = 2:1, 70%), **3** (30%); for **R = C_12_H_25_**, (a) TFA (50% aq.), r.t., 1.5 h; (b) Ac_2_O, py, 40 min, to give only **5**, 43%, α/β = 1:0.3, 2 steps or (a) TFA (65% aq.), r. t., 2.5 h; (b) Ac_2_O, py, 1 h, **5** (α/β = 1:0.3, 9%), **6** (21%).

**Scheme 2:**
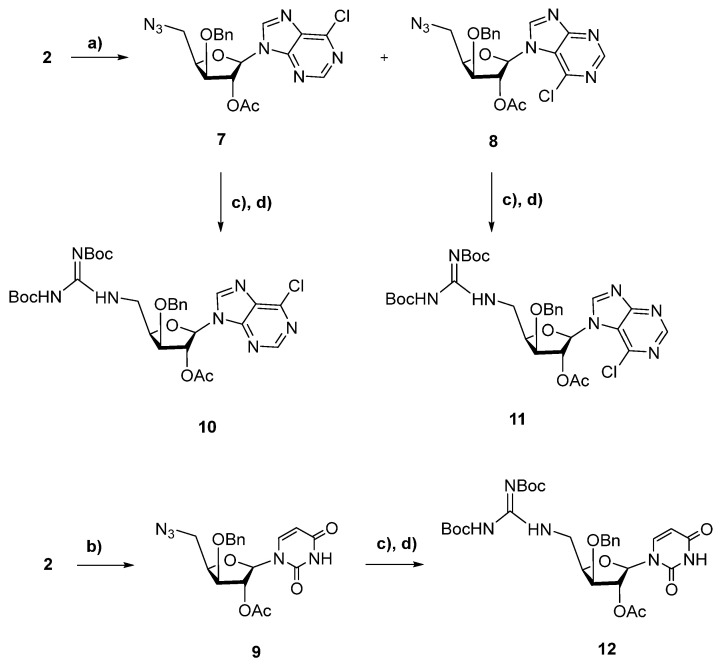
**Reagents and Conditions:** (a) 6-Cl-purine, BSA, TMSOTf, CH_3_CN, 65 °C, 4 h, 75% (**7**), 10% (**8**); (b) uracil, BSA, TMSOTf, CH_3_CN, 65 °C, 24 h, 58%; (c) PPh_3_, THF/H_2_O (10/1), r. t., 3 h; (d) *N*,*N*′-diBoc-*N*′′-triflylguanidine, DIPEA, AcOEt, r. t., 5 h, 18% (**10**), 46% (**11**), 46% (**12**), 2 steps.

The 5-azido-3*-O*-benzyl xylofuranose derivative **1** [22] was the precursor used in the synthesis of the target nucleosides. It was converted into the corresponding glycosyl acetate donor **2** via trifluoroacetic acid-mediated hydrolysis followed by acetylation using acetic anhydride in pyridine (Scheme 1). Treatment of **1** with aqueous TFA for 30 min and further acetylation led to compound **2** as sole product. On the other hand, an increase in acid hydrolysis time to 2 h led also to the δ-glyconolactam-type iminosugar **3** as a secondary product, which was obtained in a significant yield (30%) along with **2** (70%). The formation of the latter compound can be explained by an intramolecular Schmidt–Boyer reaction [34] of the intermediate 5-azido aldopentose. Such transformation had already been reported for the treatment of δ-azido aldoses comprising acid-labile protecting groups under protic or Lewis acidic conditions [35]. To additionally evaluate the scope of this conversion, similar conditions were applied to the 3-*O*-dodecyl analog **4** [23], also affording the δ-xylonolactam (**6**), which was, in this case, the major product. The lactam structure of **3** and **6** was unambiguously confirmed by NMR spectroscopy, namely, by the appearance of the signals corresponding to the lactam carbonyl carbon at *δ* 166.9 and 167.9 ppm in their ^13^C NMR spectra, which correlate with H-2 and H-5a signals in their HMBC spectra. In both cases, separation of the xylosyl acetate (**2** or **5**) from its xylonolactam counterpart (**3** or **6**) was achieved by column chromatography. Nucleosidation of the 3′-*O*-benzyl xylofuranosyl acetate **2** was then performed with pre-silylated 6-chloropurine and uracil in the presence of trimethylsilyl triflate (TMSOTf) in acetonitrile at 65 °C (Scheme 2). Using 6-chloropurine, the N^9^ and N^7^ nucleosides **7** and **8** were obtained in yields of 75% and 15%, respectively, and were separated by column chromatography. The regiochemistry of the N-glycosidic bond of **7–8** was clearly assigned based on their HMBC spectra. In the case of the N^9^-linked nucleoside **7**, a HMBC correlation between H-1′ of the xylofuranyl unit and C-4 of the purine moiety was observed, while for the N^7^ regioisomer **8**, a correlation between H-1′ and C-5 was displayed. N-Glycosylation of uracil afforded the N^1^ nucleoside **9** as a sole product in 58% yield, a similar outcome to that previously reported when this reaction was performed under microwave irradiation (MW) [22]. However, using conventional heating, as in this case, a considerably longer time was needed for reaction completion (24 h vs. 45 min using MW). The azido nucleosides **7–9** were then subjected to reduction of their azide groups using the Staudinger method (triphenylphosphine in tetrahydrofuran (THF)/water), which was followed by guanidinylation of the thus-obtained (non-isolated) amino nucleosides with *N*,*N*′-bis(*tert*-butoxycarbonyl)-*N*″-triflylguanidine in the presence of diisopropylethylamine (DIPEA), using a similar protocol to that previously implemented by us [29]. This sequential Staudinger/guanidinylation protocol led to the corresponding guanidino nucleosides **10–12** in modest (18%) to moderate yields (46%).

### 2.2. Biological Evaluation

Aiming at inspecting their bioactivity profile, the nucleosides **7–12** and the xylonolactam **3** were evaluated for their cytotoxicity against cancer cells, for their ability to inhibit the activity of cholinesterases, and for their neuroprotective effects. The significantly active compounds were also evaluated for their in vitro hepatotoxicity.

#### 2.2.1. Cholinesterase Inhibition Assays

The inhibition of cholinesterases (ChEs), enzymes that hydrolyse the neurotransmitter acetylcholine (ACh), is still a major therapeutic strategy for symptomatic treatment in the mild and moderate stages of Alzheimer’s disease.

While AChE is more abundant than BChE in the brain tissues of the AD patients, its activity is significantly reduced during the progression of the disease, whereas that of BChE increases. Therefore, both ChEs are important targets, the inhibition of which prevents the hydrolytic degradation of ACh, therefore ameliorating the deficient brain cholinergic neurotransmission of AD patients [24]. Moreover, it has been reported that selective BChE inhibition lowers beta-amyloid peptide levels in rodent brain and in cultured human SK-N-SH neuroblastoma cells without affecting cell viability [36].

Apart from its cholinergic role, there is increasing evidence that ACh acts as a growth factor for cell proliferation in various types of cancers, and, therefore, enzymes that regulate the ACh pathway, namely, cholinesterases, may play a role in tumorigenesis [25,26,37,38]. Indeed, various examples of molecules that inhibit AChE or BChE, among which are the anti-AD drugs galantamine [39] and donepezil [40], have shown in vitro anticancer effects [41,42]. In turn, some anticancer drugs, such as doxorubicin, daunorubicin [43], irinotecan [44], and sunitinib [45], were reported to exhibit AChE inhibitory abilities. There are however ambiguous findings regarding the contribution of AChE and BChE for cancer, since in some cancer cells these enzymes are overexpressed, while, in others, their activity is downregulated [26,46,47,48]. Moreover, these changes to ChE expression appear to be also related to the tumoral stage [26,47,49]. Therefore, since ChE expression is cancer-type specific, anticancer therapeutic strategies targeting cholinesterases may either involve ChE inhibition or enhancing ChE expression.

The inhibitory effects of the compounds on AChE and BChE were assessed by Elman’s method. Galantamine, a clinically used ChE inhibitor, was used as a reference. The significant results are displayed in Table 1.

The significant ChE inhibitors were the 5′-guanidino xylofuranosyl purine nucleosides **10** and **11.**

**Figure 4:**
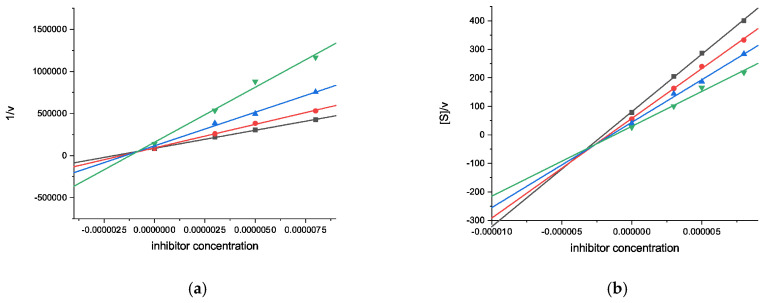
Dixon (**a**) and Cornish–Bowden (**b**) plots for the inhibition of BChE by compound **10**.

**Figure 5:**
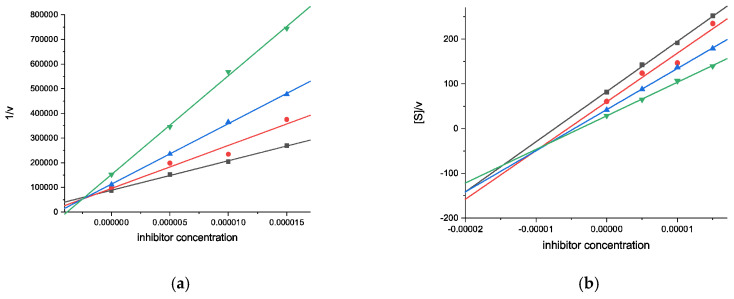
Dixon (**a**) and Cornish–Bowden (**b**) plots for the inhibition of BChE by compound **11**.

While galantamine is a competitive inhibitor of both ChEs, with selectivity for AChE (*K*_i_ = 0.54 ± 0.01 μM), compounds **10** and **11** were selective towards BchE with inhibition constants *K*_i_ in the submicromolar and micromolar order of concentration, respectively. Both exhibited a mixed-type inhibition with a competitive component that is more pronounced than the uncompetitive one (*K*_i_ < *K*_i_′). The N^9^ nucleoside **10** showed a higher inhibitory effect (*K*_i_ = 0.89 ± 0.08 μM, *K*_i_′ = 2.96 ± 0.39, Figure 4) than its N^7^ congener **11** (*K*_i_ = 2.34 ± 0.20 μM, *K*_i_′ = 10.6 ± 1.4 μM, Figure 5). Their inhibitory activities towards BchE were ca. 10-fold (**10**) and 4-fold higher (**11**) than that of galantamine.

#### 2.2.2. Neuroprotective Capacity on a Parkinson’s Disease Cellular Model

Parkinson’s disease (PD) is a progressive neurodegenerative disease characterized by gradual deterioration of dopaminergic neurons in the substantia *nigra pars compacta* (SNpc), to which oxidative stress has been considered to be strongly related [50]. A commonly used valuable tool to induce models of PD is the synthetic neurotoxin 6-hydroxydopamine (6-OHDA), which reproduces major cellular events related to this disease, including oxidative stress, neuroinflammation, neurodegeneration, and apoptotic neuronal cell death [51].

The cytotoxic activity of compounds **3**, **7–12** (0.01–10 µM; 24 h) was firstly evaluated to determine their non-toxic concentrations. Only at these non-toxic concentrations (0.01–10 µM; 24 h) were the compounds subsequently tested for their neuroprotective potential in an in vitro PD damage model using differentiated SH-SY5Y neuroblastoma cells. The cells, previously differentiated to obtain a dopaminergic neuronal phenotype [52], were treated with compounds in the presence of the neurotoxin 6-OHDA (100 µM). The cells’ viability was estimated using the 3-(4,5-dimethylthiazol-2-yl)−2,5-diphenyltetrazolium bromide (MTT) assay.

**Figure 6:**
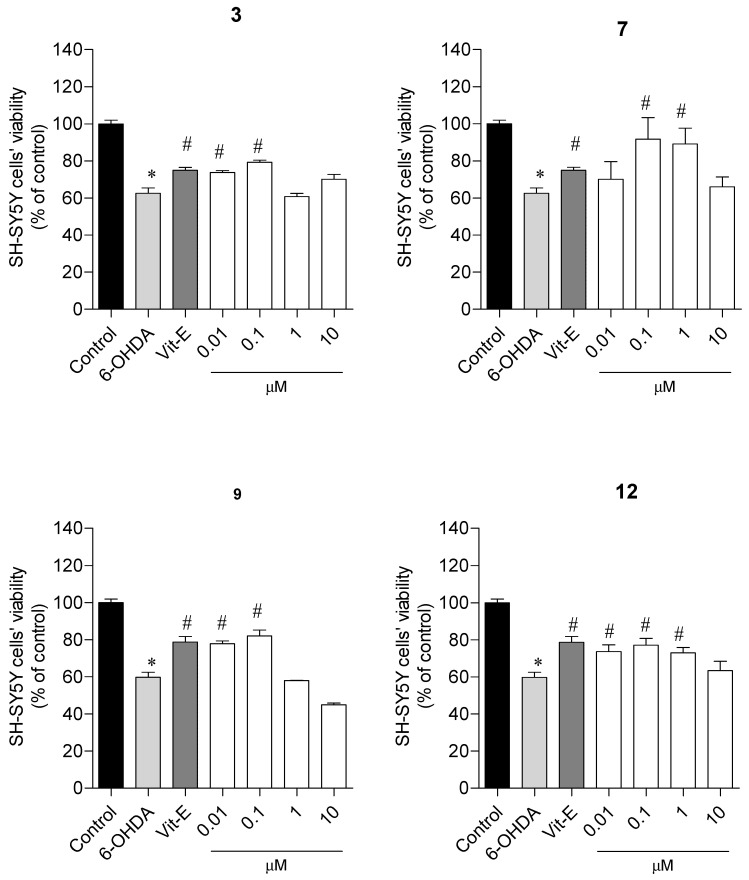
Neuroprotective effects of compounds **3**, **7**, **9**, and **12** (0.01–10 μM) on differentiated SH-SY5Y cells when exposed to 6-OHDA (100 µM) for 24 h. The viability cells percentage was estimated by the MTT assay and represented as the mean ± SEM for 3 independent experiments. Vitamin E was used as positive control (vitamin E (Vit-E)—25 µM). Values are expressed as a percentage of (*) control cells and compared to cells treated with 6-OHDA (#) alone by ANOVA, Dunnett’s test *p* < 0.05.

As shown in Figure 6, the exposure of differentiated SH-SY5Y cells to 6-OHDA (100 μM) reduced cells’ viability by 40%. However, the treatment with the xylonolactam **3**, the N^9^-linked 5′-azido 6-chloropurine nucleoside **7**, the 5′-azido uracil nucleoside **9**, and the 5′-guanidino uracil nucleoside **12** (0.01–1 μM) promoted a significant recovery from the neurotoxicity induced by the neurotoxin, increasing SH-SYSY cells’ viability by approximately 20% (18.1 ± 3.7%; 19.6 ± 5.9%; 18.6 ± 2.4%; 17.0 ± 3.5%, respectively) when compared to 6-OHDA alone (61.3 ± 1.4% of viable cells). The other compounds did not show a significant ability to neutralize 6-OHDA-induced toxicity (Figure S8; see Supplementary Materials).

#### 2.2.3. Cytotoxicity on Different Malignant Cell Lines

The in vitro cytotoxic activity of compounds **3** and **7**–**12** on human cancer cell lines, namely, HCT-15 (colorectal adenocarcinoma), MCF-7 (breast adenocarcinoma), and DU-145 (prostate adenocarcinoma), the human SH-SY5Y neuroblastoma cell line, as a model for a neuronal cell [53], and non-malignant murine hepatocytes FL83B was evaluated by MTT assay (Table 2).

Among 5′-azido nucleosides, the N^9^-linked 6-chloropurine nucleoside **7** was the sole compound that revealed cytotoxic activity, with selectivity against the colorectal cancer cell (HCT-15). It exhibited higher cytotoxicity against the HCT-15 cell line than the positive control, 5-fluorouracil, displaying an IC_50_ value two-fold lower than this reference anticancer drug. It exhibited an IC_50_ value of 22.74 μM in the hepatocyte cell line FL83B. Its 5′-guanidino N^9^-linked 6-chloropurine nucleoside derivative **10** was selective to the prostate cancer cell (DU-145), displaying an IC_50_ value of 27.63 μM, and showed relatively low in vitro hepatotoxicity in FL83B cells (IC_50_ = 73.74 μM). On the other hand, the 5′-guanidino N^7^-linked 6-chloropurine nucleoside counterpart of the former (**11**) was the most active compound of the series, possessing cytotoxicity against all tested cells with IC_50_ values ranging from 64.07 (HCT-15 cells) to 12.14 μM (SH-SY5Y cells). Its activity against HCT-15 cells was ca. 2.4-fold higher than that exhibited by 5-fluorouracil and was lower that than exhibited towards breast cancer MCF-7 cells (IC_50_ = 43.67 μM) and prostate cancer DU-145 cells (IC_50_ = 24.48 μM). Noteworthy to mention is that the cytotoxicity of compound **11** in MCF-7 cells is only 1.6-fold lower than that of the reference drug tamoxifen, while its effect against DU-145 cells is 4-fold higher than that of cisplatin. Its hepatotoxicity (FL83B cells, IC_50_ = 24.08 μM), although higher than that of 5-fluorouracil, is similar to that exhibited by cisplatin (IC_50_ = 25.71 μM) and slightly lower than that of tamoxifen (IC_50_ = 19.51 μM). The 5′-guanidino uracil nucleoside **12** was selective towards HCT-15 cells (IC_50_ = 76.02 μM), exhibiting a ca. 2-fold higher cytotoxicity against this cell line than the reference drug.

### 2.3. Computacional Studies

#### 2.3.1. Molecular Docking Studies

To give an insight on the binding interactions on a molecular level into the competitive component of the BChE inhibitory activities of compounds **10** and **11** and to understand the higher activity exhibited by the regiosomer N^9^, molecular docking simulations (MOE 2020 0901) were performed using the crystal structure of BChE (PDB: 4BDS), which was retrieved from the RCSB Protein Data Bank.

**Figure 7:**
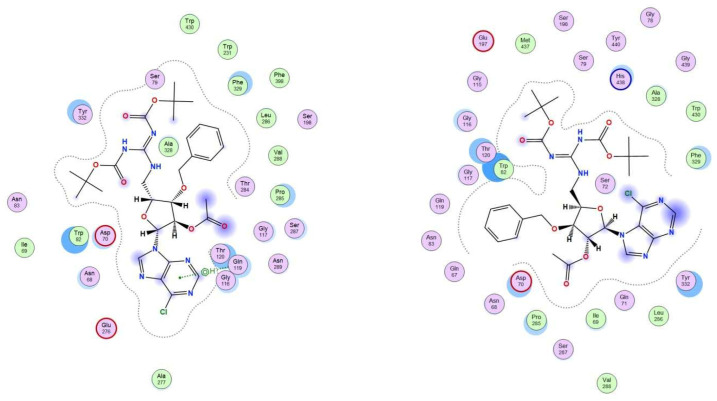
2D Schematic molecular interactions of the lowest-energy docking poses for compound **10** (**left**) and **11** (**right**) in BChE.

The 2D visualization illustrates the molecular interactions of compounds **10** and **11** within the enzyme binding site (Figure 7). It highlights key interactions, including steric hindrance (marked in blue), affecting binding efficiency as well as arene-H- interactions.

**Figure 8:**
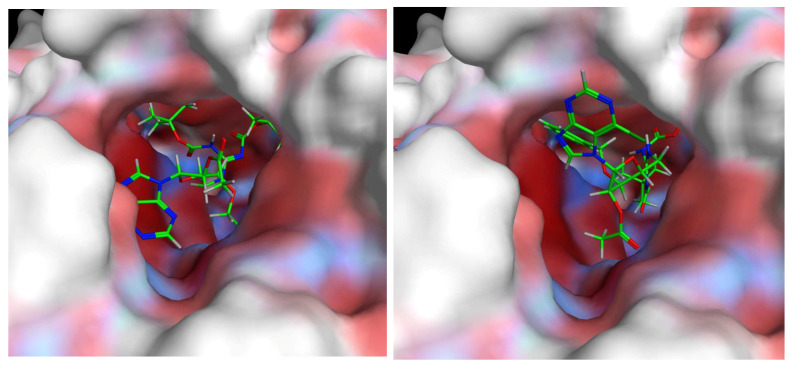
3D Representations of the lowest-energy docking poses for compound **10** (**left**) and **11** (**right**) in BChE. The surface of the binding pocket was colored by hydrophobicity (blue: polar, red: hydrophobic).

The 3D representation (Figure 8) shows the spatial positioning of both compounds within the enzyme pocket, with the surface colored to indicate polar regions (blue) and hydrophobic areas (red).

The N^9^-nucleoside **10** establishes arene-H interactions with Gln 119, suggesting a favorable binding mode that likely contributes to its inhibitory activity. In contrast, its N^7^-linked regioisomer **11** lacks this interaction and instead shows steric hindrance due to the purine heterocycle. This structural interference may explain its lower inhibition efficiency, aligning well with the experimental results.

#### 2.3.2. In Silico log *p* Prediction

The 1-octanol/water partition coefficient (*p*) of a compound, commonly represented by the log *p* value, is a measure of its lipophilicity, which is a key physicochemical property that directly affects its pharmacokinetic properties. The compound’s absorption, distribution in the body, plasma protein binding, ability to cross cell membranes and biological barriers, including crossing the blood-brain barrier and ability for tissue penetration, metabolism, clearance from the body, and toxicity (ADMET properties) are dictated by its liphophilicity [54]. According to the “Lipinski’s Rule of 5”, a compound to be orally bioavailable should have a log *p* value ≤ 5.

Aiming at the evaluation of the drug-likeness of the active nucleosides in this context, particularly their potential to cross membrane barriers, in silico calculations of their logPs were made by using ALOGPS 2.1 (Table 3) [55,56].

All synthesized compounds have a calculated log *p* ≤ 5, which indicate their drug-likeness according to the Lipinski rule of 5. The predicted log *p* values (1 < log *p* < 5) also show the propensity of these compounds for cell penetration. The c log *p* value for the xylonolactam **3** falls within the optimal interval for good oral bioavailability and intestinal absorption [57]. The calculated lop *p* values for the azido nucleosides **7**, **8**, and **9** and for the guanidine nucleoside **12** are within the ideal range for gastric absorption [58] and for targeting the central nervous system (CNS), i.e., for crossing the blood–brain barrier (BBB) [59].

Although the lipophilicities of 5′-guanidine 6-choloropurine nucleosides are the highest among the active compounds, their predicted log *p* values are still acceptable for CNS targeting since they are in accordance with the minimal hydrophobicity (*c* log *p* < 5) required for CNS targeting and, hence, BBB-penetrating drugs [60].

## 3. Conclusions

Novel types of 5′-functionalized nucleosides, namely, 5′-guanidino 6-chlopurine and uracil nucleosides based on a 3-*O*-benzyl xylofuranosyl moiety, were synthesized in five steps from 5′-azido 3-*O*-benzyl xylofuranose. Key synthetic steps included nucleobase *N*-glycosylation and Staudinger reduction/guanidinylation of the obtained azido nucleosides.

The synthesized guanidino nucleosides showed significant biological activities pointing out their anti-neurodegenerative disease potential and/or anticancer interest.

The 5′-guanidino 6-chloropurine nucleosides **10** and **11** revealed a strong and selective inhibitory ability towards butyrylcholinesterase (BChE), with a higher effect for the N^9^-linked regioisomer **10** (*K*_i_ = 0.89 ± 0.08 μM, *K*_i_′ = 2.96 ± 0.39 μM). Moreover, nucleoside **10** did not show significant cytotoxic activity towards human neuroblastoma cells (SH-SY5Y, IC_50_ > 100 μM) and exhibited rather low cytotoxicity to FL83B hepatocytes (IC_50_ = 73.74 μM). These results indicate the interest in nucleoside **10** as a promising lead compound for Alzheimer’s disease symptomatic treatment, allying its significant ability to inhibit BChE with its low in vitro neuro- and hepatotoxicity.

The parent 5′-guanidino N^7^ -linked nucleoside **11** was shown to inhibit this cholinesterase with a *Ki* value that is ca. 2-fold higher than that of the N^9^-counterpart, while it showed higher cytotoxicity to hepatocytes (IC_50_ = 24.08 μM) and to the neuroblastoma cells (IC_50_ = 12.14 μM).

These findings reinforce the interest shown in previous reports [15,16,17,18,29] of exploring nucleoside analogs in the search for new lead cholinesterase inhibitors, further highlighting the anticholinesterase potential of guanidino derivatives [29].

Moreover, the predicted lipophilicities of these compounds (c log *p* ~ 4.5) indicate their propensity for BBB penetration and for CNS targeting, which, additionally, show the interest in them as potential anti-AD agents and their suitability for further investigations, including in vivo (model) studies.

Concerning the in vitro anticancer evaluation, all tested guanidine nucleosides emerged as broad or selective *hit* anticancer molecules. The N-glycosidic bond regiochemistry in the guanidino purine nucleosides also resulted in differences regarding their cytotoxic activities in cancer cells. While the N^9^-nucleoside **10** was selective to prostate cancer cells (DU-145, IC_50_ = 27.63 μM), its N^7^-regioisomer **11** displayed broad anticancer activity, affecting the viability of all cell lines tested. The in vitro hepatocytotoxicity of nucleoside **11**, although higher than that of its N^9^-congener, is similar to or lower than those of the clinically used anticancer drugs cisplatin and tamoxifen. The 5′-guanidino N^7^ -linked 6-chloropurine nucleoside (**11**) as well as the uracil nucleoside **12** showed **a** ca. 2-fold higher cytotoxic effect towards the colorectal cancer cells HCT-15 than the reference drug 5-fluorouracil (5-FU). The 5′-guanidino uracil nucleoside was, however, selectively active against this cancer cell and showed no significant in vitro hepatotoxicity, thus outperforming 5-FU. The former nucleoside also showed moderate neuroprotective effects in the SH-SY5Y-based Parkinson’s disease (PD) cellular model, with a ca. 20% increase in the viability of the damaged cells. The calculated log *p* values of the guanidine nucleosides (2.5 to 4.5) are indicative of their ability to penetrate cell membranes and show their potential for exhibiting better pharmacokinetic properties than the reference anticancer drugs.

Noteworthy to highlight are the dual selective BChE inhibition/anticancer activities of the nucleosides **10** and **11**, which make them promising dual-indication lead compounds for both AD and cancer. The N^9^ nucleoside **10** can be emphasized, due to its rather low neuro- and hepatotoxicity.

Although the participation of cholinesterases in cancer remains to be clarified and established, a link between the BChE and the anticancer activities of compounds **10** and **11** cannot be discarded. In particular, alterations to the expression of BChE in prostate cancer DU-145 cells have been reported and evidence has been found to support that BChE upregulation is associated with cancer recurrence [49].

Concerning the 5′-azido 3-*O*-benzyl xylofuranosyl nucleosides, the N^9^-linked chloropurine nucleoside **7** was the sole significantly active compound, revealing cytotoxicity against the colorectal cancer cell line with an IC_50_ 2-fold lower than that of 5-FU, although displaying higher cytotoxicity to FL83B hepatocytes than the former (IC_50_ = 22.74 μM). Moreover, the predicted lipophilicity of this compound (c log *p* = 2.18) makes it more appropriate for a potential therapeutic application than the reference drug 5-fluorouracil (c log *p* = −0.58). This nucleoside also revealed a moderate neuroprotective effect in the PD cellular model.

It is worth mentioning that in line with our previous findings [17,22], the inclusion of a 3-*O*-benzyl xylofuranosyl unit in nucleoside analogs leads to molecules possessing significant bioactivities.

In these molecules, the lack of a 5′-hydroxyl group precludes them from being phosphorylated by kinases and converted into nucleoside triphosphate metabolites for further recognition by DNA polymerases and the inhibition of DNA chain elongation. Therefore, their cytotoxic effects likely arise from a different mechanism of action than that exhibited by the clinically used anticancer nucleosides [1,2,3]. Further investigations will, therefore, address studying the mechanisms of action of the compounds.

The bioactivity results herein presented clearly show the increased biological potential of guanidino nucleosides relative to their azido counterparts, with compounds showing significant cytotoxicity to cancer cells, the ability to inhibit butyrylcholinesterase, and neuroprotective effects, among which are promising lead/hit molecules. These findings demonstrate the interest in installing guanidine moieties in nucleosides in the search for new therapeutic agents.

## 4. Experimental Section

### 4.1. Chemistry

#### 4.1.1. General Methods

Chemical reactants were purchased from Sigma-Aldrich, Alfa Aesar and TCI. The reactions were followed by TLC using Merck 60 F254 silica gel aluminium plates. Spots were visualized under UV light (254 nm) and/or after immersion in a 10% (*v*/*v*) metanolic H_2_SO_4_ solution followed by charring with a heat gun (200 °C). Compounds were purified by flash column chromatography on silica gel 60 G (0.040–0.063 mm, Merck. NMR experiments were carried out using a BRUKER Avance 400 spectrometer operating at 400.13 MHz (for ^1^H NMR) and 100.62 MHz (for ^13^C NMR). Spectra in CDCl_3_ were referenced to the solvent peak [7.26 ppm for ^1^H NMR (C*H*Cl_3_ in CDCl_3_) and 77.16 ppm for ^13^C NMR)]. Two-dimensional NMR experiments (COSY, HSQC, and HMBC) were performed for supporting signal assignment. Chemical shifts are given in parts per million (ppm) and coupling constants (*J*) are reported in Hertz (Hz). Mass spectrometry analysis was performed using The Bruker Imapct II UHR–QqTOF (Bruker Daltonics) instrument with electrospray ionization (ESI) with the following parameters: Capillary voltage was set at 4500 V, nebulizer was set to 2 bar, drying gas flow rate was 6.0 L/min, and drying temperature was 200 °C. The acquisition was performed in the positive ionization mode at spectra rate of 1 Hz, in the mass range of 50 to 1500 *m*/*z*. The collision energy was set to 10 eV, with a transfer time 60 ms. The spectra were externally calibrated by infusion of the 1μM sodium formate-acetate adducts across all mass ranges (infusion flow 180 µL/h). Calibration of MS files was performed using Compass Data Analysis 6.3 (Bruker, Daltonics) in high-precision calibration mode (HPC) giving an error of <0.2 ppm. Optical rotations (589 nm, sodium D line, 20 °C) were measured using an Anton Paar MCP100 Polarimeter.

#### 4.1.2. General Procedure for TFA-Mediated Acetonide Hydrolysis of 5-Azido 3-O-Protected-5-Deoxy-1,2-O-Isopropylidene-α-D-Xylofuranose and Further Acetylation

A solution of 5-azido 3-O-alkyl-5-deoxy-1,2-O-isopropylidene-α-D-xylofuranose (1, 4, 1 mmol) in aqueous trifluoroacetic acid soln (65% aq., 8.5 mL) was stirred at room temp. for 30 min–2 h. The solvents were co-evaporated with toluene and the resulting residue was dried under vacuum and then dissolved into a mixture of acetic anhydride (8.5 mL) and pyridine (10 mL). The solution was stirred at 40 min–1 h. After co-evaporation of the solvents with toluene, the crude was subjected to column chromatography on silica gel.

According to this procedure, and with a reaction time of 30 min for the hydrolysis step of 5-azido-3-*O*-benzyl-5-deoxy-1,2-*O*-isopropylidene-a-d-xylofuranose (**1**) [19], followed by acetylation, 1,2-di-*O*-acetyl-5-azido-3-O-benzyl-5-deoxy-α-D-xylofuranose (**2**) was obtained in 80% yield [22], while a reaction time of 2 h for the hydrolysis step of **1** and further acetylation led to **2** (70%, 2 steps, α/β mixture, 2:1, colorless oil) and **3** (30%, 2 steps, colorless crystals).

Separation of **2** from **3** was achieved by column chromatography (hexane/EtOAc, from 1:1 to 1:3).

Starting from 5-azido-5-deoxy-3-O-dodecyl-1,2-O-isopropylidene-α-D-xylofuranose (**4**) [23], a 1.5 h for the hydrolysis step, followed by acetylation, led to 1,2-di-*O*-acetyl-5-azido-5-deoxy-3-*O*-dodecyl-α,β-D-xylofuranose (**5**, 43%, α/β mixture, 1:0.3, 2 steps, colorless oil), while a reaction time of 2.5 h for the hydrolysis step led, after acetylation, to **5** (9%, α/β mixture, 1:0.3, 2 steps) and **6** (21%, colorless oil). Separation of **5** from **6** was achieved by column chromatography (hexane/EtOAc, 8:1, then 4:1, then 1:2).

Data for **2** and **5** were previously reported [22,23].

**Data for *N*-acetyl 2,4-di-O-acetyl-3-O-benzyl-D-xylono-1,5-lactam (3):** *R*_f_ = 0.17 (hexane/EtOAc, 4:1). ${\left[\alpha \right]}_{D}^{20}$ = + 40.2 (c = 1.0, in CHCl_3_). ^1^H NMR (CDCl_3_, 400 MHz): *δ*= 7.40–7.28 (m, 5 H, Ph), 5.37 (d, H-2, *J*_2,3_ = 7.9); 5.19 (ddd, H-4); 4.81–4.73 (m, 2 H, H-5a, H-a from C*H*_2_Ph, *J*_3,5a_ = 1.5, *J*_4,5a_ = 3.4, *J*_5a,5b_ = 15.0), 4.68 (d, H-b from C*H*_2_Ph, *J* = 12.1), 3.76 (dt, H-3, *J_3_*_,4_ = *J_3_*_,5a_ = 1.5), 3.54 (dd, H-5_b_, *J*_4,5b_ = 1.5), 2.55 (s, C*H*_3_, NHAc), 2.18, 2.02 (2 s, 2 × C*H*_3_, OAc) ppm. ^13^C (NMR, 100 MHz): *δ* = 171.8 (*C*O, NHAc), 170.0, 169.7 (2 × *C*O, OAc), 166.9 (*C*O, lactam), 137.1 (Cq, Ph), 128.6, 128.3, 128.2 (C*H*, Ph), 79.0 (C-3); 74.1 (C-2); 72.4 (*C*H_2_Ph); 70.0 (C-4); 41.2 (C-5), 27.1 (*C*H_3_, NHAc), 21.0, 20.7 (2×*C*H_3_, OAc) ppm.

**Data for *N*-Acetyl 2,4-di-O-acetyl-3-O-benzyl-D-xylono-1,5-lactam (6)**: *R*_f_ = 0.40 (hexane/EtOAc, 4:1). ${\left[\alpha \right]}_{D}^{20}$ = −2.9 (c = 0.35, in CHCl_3_). ^1^H NMR (CDCl_3_, 400 MHz): *δ*= 6.48 (s, 1 H, N*H*), 5.20 (d, H-2, *J*_2,3_ = 7.2); 5.04 (ddd, H-4), 3.73 (dd, 1 H, H-3, *J*_3,4_ = 5.1), 3.67–3.53 (m, 3 H, H-a, H-b from C*H*_2_-1′, H-5a), 3.31 (dt, 1 H, H-5 b, *J*_5a,5b_= 13.5, *J*_4,5_ = *J*_5b,NH_ = 4.5), 2.18, 2.09 (2 s, 2 × C*H*_3_, OAc), 1.58–1.47 (m, 2 H, C*H*_2_-2′), 1.37–1.19 (br.s, 18 H, C*H*_2_-3′ to C*H*_2_-11′), 0.87 (t, 1 H, C*H*_3_-12, *J* = 6.3) ppm. ^13^C (NMR, 100 MHz): *δ* = 170.0, 170.0 (2 × *C*O, OAc), 167.9 (*C*O, lactam), 78.8 (C-3), 71.9 (C-2), 71.6 (*C*H_2_-1′), 69.8 (C-4), 42.0 (C-5), 32.0, 29.9, 29.8 29.8, 29.7, 29.5, 29.5, 26.1 (*C*H_2_-2′ to *C*H_2_-11′), 21.1, 20.9 (2×*C*H_3_, OAc), 14.3 (*C*H_2_-12′) ppm. HRMS: calcd for C_21_H_37_NO_6_ [*M* + H]^+^ 400.2694, found 400.2695.

**9-(2-O-Acetyl-5-azido-3-O-benzyl-5-deoxy-β-D-xylofuranosyl)-6-chloropurine** (7) and 7-(2-O-acetyl-5-azido-3-O-benzyl-5-deoxy-β-D-xylofuranosyl)-6-chloropurine (8): To a solution of 6-chloropurine (502 mg, 3.3 mmol) in acetonitrile (13 mL), *N*,*O*-bis(trimethylsilyl) acetamide (BSA, 0.8 mL, 3.3 mmol) was added. The mixture was stirred at room temperature for 20 min. A solution of 1,2-di-*O*-acetyl-5-azido-3-O-benzyl-5-deoxy-α-D-xylofuranose (2, 377 mg, 1.1 mmol) in acetonitrile (13 mL) was added to the previous solution, followed by dropwise addition of trimethylsilyl triflate (TMSOTf, 1.3 mL, 7.2 mmol). The resulting mixture was stirred at 65 °C for 4 h. The solution was then diluted with CH_2_Cl_2_ and it was neutralized with sat. sodium bicarbonate soln. The aqueous phase was extracted with dichloromethane (3×) and the combined organic phases were washed with brine and then dried with anhydrous MgSO_4_. After filtration and concentration under vacuum, the residue was purified by column chromatography (hexane/EtOAc, 3:1) to afford 7 (359 mg, 75%) and 8 (48 mg, 10%) as colorless oils.

**Data for 7:** *R*_f_ = 0.43 (hexane/EtOAc, 2:1). ${\left[\alpha \right]}_{D}^{20}$ = −26.1 (c = 1.1, in CHCl_3_). ^1^H NMR (CDCl_3_, 400 MHz): *δ* = 8.73 (s, 1 H, H-2), 8.42 (s, 1 H, H-8), 7.37–7.19 (m, 5 H, C*H*, Ph), 6.37 (s, 1 H, H-1′), 5.49 (s, 1 H, H-2′), 4.70 (d, part A of AB system, 1 H, H-a from C*H*_2_Ph, *J*_a,b_ = 11.6 Hz), 4.59 (d, part B of AB system, 1H, H-b from CH_2_Ph, *J*_a,b_ = 11.6 Hz), 4.41 (ddd, 1 H, H-4′), 4.09 (d, 1 H, H-3′, *J*_3′,4′_ = 3.6 Hz), 3.75 (dd, part A of ABX system, 1 H, H-5′a, *J_5′a,5′b_* = 12.7, *J*_4′,5′a_ = 6.8 Hz), 3.65 (dd, parte B do sistema ABX, 1 H, H5′b, *J*_5′a,5′b_ = 12.7, *J*_4′,5′b_ = 6.0 Hz, 1H), 2.20 (s, 3 H, C*H*_3_, OAc) ppm. ^13^C NMR (CDCl_3_, 100 MHz): *δ* = 169.6 (*C*O, Ac), 152.3 (C-2), 151.2 (C-6), 151.2 (C-4), 143.8 (C-8), 136.0 (Cq, Ph), 131.8 (C-5), 128.9, 128.7, 128.4 (CH, Ph), 87.9 (C-1′), 81.9 (C-4′), 80.0 (C-3′), 79.5 (C-2′), 72.6 (*C*H_2_Ph), 49.3 (C-5′), 20.9 (*C*H_3_, OAc) ppm. HRMS: calcd for C_19_H_18_ClN_7_O_4_ [*M* + Na]^+^ 466.1001, found 466.1042.

**Data for 8**: *R*_f_ = 0.19 (hexane/EtOAc, 2:1). ${\left[\alpha \right]}_{D}^{20}$ = + 57.4 (c = 0.6, in CHCl_3_). ^1^H NMR (400 MHz, CDCl_3_): *δ* = 8.90 (s, 1H, H-2), 8.63 (s, 1H, H-8), 7.29–7.13 (m, 5 H, C*H*, Ph), 6.63 (s, 1H, H-1′), 5.40 (s, 1H, H-2′), 4.62 (d, part A of AB system, 1 H, H-a from C*H*_2_Ph, *J_a,b_* = 11.8 Hz), 4.55 (d, part B of AB system, 1 H, H-b from CH_2_Ph, *J_a,b_* = 11.8 Hz), 4.43 (ddd, 1 H, H-4′), 4.05 (d, 1 H, H-3′, *J*_3′,4′_ = 3.3 Hz), 3.81 (dd, part A of ABX system, 1H, H-5′a, *J*_5′a,5′b_ = 12.7, *J*_4′,5′a_ = 6.9 Hz), 3.68 (dd, part B of ABX system, 1 H, H-5′b *J*_5′b,5′a_ = 12.7, J_4′,5′b_ = 6.0 Hz), 2.20 (s, 3 H, CH_3_, OAc) ppm. ^13^C NMR (101 MHz, CDCl3): *δ* = 169.4 (CO, OAc), 162.6 (C-4), 152.7 (C-2), 147.3 (C-8), 142.3 (C-6), 136.0 (Cq, Ph), 128.8, 128.6, 128.5 (CH, Ph), 121.6 (C-5), 90.4 (C-1′), 82.4 (C-4′), 80.1 (C-3′), 79.9 (C-2′), 72.7 (CH_2_, Ph), 49.1 (C-5′), 20.8 (CH_3_, OAc) ppm. HRMS: calcd for C_19_H_18_ClN_7_O_4_ [*M* + Na]^+^ 466.1001, found 466.1019.

**1-(2-*O*-Acetyl-5-azido-3-*O*-benzyl-5-deoxy-**β**-D-xylofuranosyl)uracil (9)**: Compound **9** was obtained according to a similar procedure for the synthesis of **7–8**, starting from 1,2-di-*O*-acetyl-5-azido-3-*O-*benzyl-5-deoxy-α,β-D-xylofuranose (**2**, 152 mg, 0.44 mmol) and uracil (148 mg, 1.3 mmol), and using BSA (0.33 mL, 1.34 mmol) and TMSOTf (0.5 mL, 2.8 mmol). The mixture comprising silylated uracil, **2** and TMSOTf was stirred at 65 °C for 24 h. Purification by column chromatography (EtOAc/hexane, 1:1) afforded the title compound (100 mg, 58%) as a colorless oil. Data were in accordance with the previously reported data [22].

**General procedure for the Staudinger reduction-guanidinylation of azido nucleosides**: To a solution of azido nucleoside (0.02 mmol) in THF/H_2_O (2 mL, 10: 1), triphenylphosphane (2–4 equiv.) was added and the mixture was stirred at room temperature for 3 h. The solvents were evaporated, and the crude residue was dried under vacuum. The residue was then dissolved in AcOEt (2.5 mL) and to the resulting solution, under nitrogen atmosphere, *N*,*N*′-bis(tert-butoxycarboyl)-*N*′′-triflylguanidine (2–3 equiv.) and DIPEA (6 equiv.) were added. After stirring at room temp. for 5 h and concentration under reduced pressure, the crude residue was subjected to column chromatography.

**9-{2-O-acetyl-3-O-benzyl-5-deoxy-5-[*N*′,*N*′′-bis(*tert*-butoxycarbonyl)]guanidino-β-D-xylofuranosyl}-6-chloropurine (10)**: Obtained according to the general procedure, starting from 9-(2-O-acetyl-5-azido-3-O-benzyl-5-deoxy-β-D-xylofuranosyl)-6-chloropurine (**7**, 155 mg, 0.35 mmol) and using triphenylphosphane (180 mg, 0.69 mmol), *N*,*N*′-bis(*tert*-butoxycarboyl)-*N*″-triflylguanidine (271 mg, 69 mmol) and DIPEA (0.36 mL, 2.07 mmol). Purification by column chromatography (hexane/AcOEt, from 4:1 to 1:1) afforded 10 (42 mg, 18%) as a yellowish oil. *R*_f_ = 0.67 (hexane/EtOAc, 1:1).${\left[\alpha \right]}_{D}^{20}$ = + 24.7 (c = 0.7, in MeOH). ^1^H NMR (400 MHz, CDCl_3_): *δ* = 11.48 (s, 1 H, N*H*Boc), 8.76 (t, 1 H, N*H*, J = 5.3), 8.72 (s, 1 H, H-2), 8.48 (s, 1 H, H-8), 7.35–7.19 (m, 5 H, C*H*, Ph), 6.35 (s, 1 H, H-1′), 5.45 (s, 1 H, H-2′), 4.68 (d, part A of AB system, 1 H, H-a from CH_2_Ph, *J_a,b_* = 11.5 Hz), 4.62 (d, part B of AB system, 1 H, H-b from CH_2_Ph, *J_a,b_* = 11.5 Hz), 4.52–4.45 (ddd, 1 H, H-4′), 4.17–4.05 (m, 2 H, H-3′, H-5′a, *J*_3′,4′_ = 3.2), 3.82 (ddd, 1 H, H-5b′, *J_5a′,5b′_* = 13.9, *J_4′,5b′_* = 6.5 Hz, *J*_5′b,NH_ = 5.3), 2.18 (s, 3 H, CH_3_, OAc), 1.50 (s, 9 H, 3 x CH_3_, Boc), 1.48 (s, 9 H, 3 x CH_3_, Boc) ppm. ^13^C NMR (101 MHz, CDCl3): *δ* = 169.6 (*C*O, OAc), 163.5 (*C*O, =NBoc), 156.6 (Cq, GN), 153.19 (*C*O, NHBoc), 152.2 (C-2), 151.2, 151.1 (C-4, C-6), 144.1 (C-8), 136.1 (Cq, Ph), 131.8 (C-5), 128.8, 128.6, 128.4 (CH, Ph), 87.9 (C-1′), 83.6 (Cq, Boc), 81.4 (C-4′), 80.8 (C-3′), 79.7, 79.7 (C-2′, Cq, Boc), 72.72 (*C*H_2_Ph), 39.4 (C-5′), 28.4 (3 × *C*H_3_, Boc), 28.2 (3 × *C*H_3_, Boc), 21.0 (CH_3_, OAc) ppm. HRMS: calcd for C_30_H_38_ClN_7_O_8_ [*M* + Na]^+^ 682.2363, found 682.2363.

**7-(2-O-acetyl-3-O-benzyl-5-deoxy-5-[*N*′,*N*′′-bis(*tert*-butoxycarbonyl)]guanidino-β-D-xylofuranosyl)-6-chloropurine (11):** Obtained according to the general procedure, starting from 7-(2-O-acetyl-5-azido-3-O-benzyl-5-deoxy-β-D-xylofuranosyl)-6-chloropurine (**8**, 30 mg, 0.07 mmol) and using triphenylphosphane (35 mg, 0.13 mmol), *N*,*N*′-bis(*tert*-butoxycarboyl)-*N*″-triflylguanidine (53 mg, 0.14 mmol) and DIPEA (0.07 mL, 0.40 mmol). Purification by column chromatography (hexane/AcOEt, 3:1) afforded **11** (20 mg, 46%) as a yellowish oil. *R*_f_ = 0.48 (hexane/EtOAc, 1:1). ${\left[\alpha \right]}_{D}^{20}$ = + 66.7 (c = 0.4, in MeOH). ^1^H NMR (400 MHz, CDCl_3_): *δ* = 11.50 (s, 1 H, N*H*Boc), 8.89 (s, 1 H, H-2), 8.83 (t, 1-H, N*H*) 8.73 (s, 1H, H-8), 7.24–7.13 (m, 5 H, C*H*, Ph), 6.60 (s, 1 H, H-1′), 5.37 (s, 1 H, H-2′), 4.58 (s, 2 H, C*H*_2_Ph), 4.50 (ddd, 1 H, H-4′), 4.15–4.03 (m, 2 H, H-5′a, H-3′, *J*_5′a,NH_ = 5.2, *J*_4′,5a′_ = 6.6 Hz, *J*_5′a,5′b_ = 14.1 Hz), 3.90 (ddd, 1 H, H-5′b, *J*_5′b,NH_ = 4.8, *J*_4′,5b′_ = 6.7 Hz, *J*_5′a,5′b_ = 14.1 Hz), 2.19 (s, 3 H, C*H*_3_, OAc), 1.52 (s, 9 H, 3× C*H*_3_, Boc), 1.49 (s, 9 H, 3 × C*H*_3_, Boc) ppm. ^13^C NMR (101 MHz, CDCl_3_): *δ* = 169.5 (CO, OAc), 163.5, 162.7 (*C*O, =NBoc, C-4), 156.7 (Cq, GN), 153.3 (*C*O, NHBoc), 152.6 (C-2), 147.7 (C-8), 142.2 (C-6), 136.2 (Cq, Ph), 128.7, 128.5, 128.4 (CH, Ph), 121.6 (Cq, C-5), 90.4 (C-1′), 83.7 (Cq, Boc), 82.0 (C-4′), 81.0 (C-3′), 80.2, 79.8 (C-2′, Cq, Boc), 72.8 (*C*H_2_Ph), 39.3 (C-5′), 28.4 (3 × *C*H_3_, Boc), 28.2 (3 × *C*H_3_, Boc), 20.9 (*C*H_3_, OAc) ppm. HRMS: calcd for C_30_H_38_ClN_7_O_8_ [*M* + Na]^+^ 682.2363, found 682.2351.

**1-(2-O-Acetyl-3-O-benzyl-5-deoxy-5-[*N*′,*N*′′-bis(*tert*-butoxycarbonyl)]guanidino-β-d-xylofuranosyl)uracil (12):** Obtained according to the general procedure, starting from 1-(2-*O*-acetyl-5-azido-3-*O*-benzyl-5-deoxy-β-d-xylofuranosyl)uracil (**9**, 75 mg, 0.19 mmol) and using triphenylphosphane (194 mg, 0.74 mmol), *N*,*N*′-bis(*tert*-butoxycarboyl)-*N*″-triflylguanidine (217 mg, 0.55 mmol) and DIPEA (0.18 mL, 1.03 mmol). Purification by column chromatography (hexane/AcOEt, 2:1) afforded **11** (53 mg, 46%) as a yellowish oil. *R*_f_ = 0.45 (hexane/EtOAc, 2:1). ${\left[\alpha \right]}_{D}^{20}$ = + 26.7 (c = 0.8, in CHCl_3_). ^1^H NMR (400 MHz, CDCl_3_): *δ* = 11.49 (s, 1 H, N*H*Boc), 9.35 (br.s, 1 H, N*H*, uracil), 8.71 (t, 1 H, N*H*, *J* = 5.3), 7.65 (d, 1 H, H-6 *J*_5,6_ = 8.1 Hz), 7.39–7.23 (m, 5 H, C*H*, Ph), 6.09 (s, 1H, H-1′), 5.60 (d, 1 H, H-5, *J*_5,6_ = 8.1 Hz), 5.17 (s, 1H, H-2′), 4.71 (d, part A of AB system, 1 H, H-a from C*H*_2_Ph, *J*_a,b_ = 11.4 Hz), 4.58 (d, part B of AB system, 1 H, H-b from C*H*_2_Ph, *J*_a,b_ = 11.3 Hz, 1 H), 4.28 (td, 1 H, H-4′, *J*_4′,5′a_ = *J*_4′,5′b_ = 6.4, *J*_3′,4′_ = 3.4 Hz, 1H), 4.00 (ddd, 1 H, H-5′a, *J*_5a′,5b′_ = 13.9, *J*_4′,5′a_ = 6.4 Hz, J_5′a,NH_ = 5.3), 3.93 (d, 1 H, H-3′, *J*_3′,4′_ = 3.4 Hz), 3.76 (ddd, 1 H, H-5′b, *J*_5′a,5′b_ = 13.9, *J*_4′,5′b_ = 6.4 Hz, 1 H), 2.13 (s, 3H, CH_3_, OAc), 1.49 (s, 9 H, 3 x CH_3_, Boc), 1.47 (s, 9 H, 3 x CH_3_, Boc).^13^C NMR (101 MHz, CDCl_3_): *δ* = 169.7 (CO, OAc), 163.5 (C-4, *C*O, =NBoc), 156.5 (Cq, GN), 153.1 (CO, NHBoc), 150.3 (C-2), 140.6 (C-6), 136.4 (Cq, Ph), 128.7, 128.6, 128.5 (CH, Ph), 102.5 (C-5), 88.8 (C-1′), 83.5 (Cq, Boc), 80.5 (C-3′, C-4′), 79.7, 79.6 (C-2′, Cq, Boc), 72.1 (CH_2_, Ph), 39.2 (C-5′), 28.3 (3 × *C*H_3_, Boc), 28.1 (3 × *C*H_3_, Boc), 20.9 (CH_3_, OAc). HRMS: calcd for C_29_H_39_N_5_O_10_ [*M* + Na]^+^ 640.2589, found 640.2592.

### 4.2. Biological Assays

#### 4.2.1. Cholinesterase Inhibition Assays

Acetylcholinesterase (from *Electrophorus electricus*), Butyrylcholinesterase (from equine serum), 5,5′-dithiobis-(2-nitrobenzoic acid) (DTNB), and acetylthiocholine iodide were purchased from Sigma.

##### Preparation of the Solutions

Preparation of 50 mM Tris-HCl buffer solutions: Tris(hydroxymethyl)-aminomethane (606 mg) was dissolved in bidestilled water (100 mL) and adjusted with HCl to a pH of 8.0 ± 0.1. Buffers were freshly prepared and stored in the refrigerator. AChE solution 2.005 U/mL: The enzyme (271 U/mg, 0.037 mg) was dissolved in freshly prepared buffer pH 8.0 (5 mL) containing NaN_3_ (0.98 mg). BChE solution 2.040 U/mL: The enzyme (7.54 U/mg, 1.353 mg) was dissolved in freshly prepared buffer pH 8.0 (5 mL) containing NaN3 (0.98 mg). DTNB solution 3 mM: DTNB (23.8 mg) was dissolved in freshly prepared buffer pH 8.0 (20 mL) containing NaCl (116.8 mg) and MgCl2 (38.0 mg). ATChI solution 15 mM: ATChI (43.4 mg) was dissolved in bidestilled water (10 mL). All solutions were stored in Eppendorf caps in the refrigerator or in the freezer, if necessary. The pure compounds were initially dissolved in DMSO; galantamine hydrobromide as standard for AChE and BChE was dissolved in bidistilled water. The final concentrations for the enzymatic assay were obtained by diluting the stock solution with bidistilled water. No inhibition was detected by residual DMSO (<0.5%).

##### Kinetic Studies

BMG Labtech Spectrostar Omega plate reader working in the slow kinetics mode and measuring the absorbance at a distinct wavelength of λ = 412 nm with center scanning mode was used for the enzymatic studies. In short, a mixture of a DTNB solution (125 µL, 3 mM in 50 mM Tris-HCl buffer, pH 8), enzyme solution (25 µL, 2 U/mL), and compounds solutions (25 µL, 3 different concentrations and water as a blank) was incubated at 30 °C for 20 min. The substrate (25 µL, [ATChI] = 0.9375 mM, 0.625 mM, 0.325 mM, 0.1875 mM) was added to start the enzymatic reaction. The absorbance data were recorded under a controlled temperature of 30 °C for 30 min at 1 min intervals at l = 412 nm. The relative inhibition was determined as the quotient of the slopes (compound divided by blank) of the linear ranges (10 repetitions). Galantamin was used as control substance. To determine the kinetic constants, standard linearization methods were used after applying an internal standard (Dixon/Cornish–Bowden plots) (triplicates).

#### 4.2.2. Neuroprotective Capacity on a PD Cellular Model

##### Differentiation of Human SH-SY5Y Neuronal Cells

SH-SY5Y cells were trypsinized (trypsin/EDTA; 0.25%) and the cell suspension was seeded in 96-well plates at a density of 2 × 10^4^ cells/well. Cells were differentiated to obtain dopaminergic neuronal phenotype according to [52], with slight modifications. After cells reached 80–85% of confluence, they were seeded in complete DMEM:F12 medium containing 10 μM retinoic acid (RA), 2.5% fetal bovine serum (FBS) and cultivated for 4 days (changed medium to the 2nd day). Ending this time, the cell medium was replaced by a new medium contained containing 10 μM RA, 80 nM 12-*O*-Tetradecanoylphorbol-13-acetate (TPA) and 2.5% FBS and cultivated for 4 days (changed medium to the 6th day). At the end of the 8th day, the cytotoxicity assay was performed.

##### Cell Viability and Neuroprotective Effects on Differentiated SH-SY5Y Cells

Cell viability on SH-SY5Y cells was evaluated after exposure to the compounds at 10 μM for 24 h. To evaluate the neuroprotective effects, cells were treated with compounds (0.01, 0.1, and 10 μM) in the presence/absence of 6-OHDA (100 μM) for 24 h. The cell viability and neuroprotective effects were assessed by a colorimetric assay based on the reduction of tetrazolium salts MTT (VWR, Solon, OH, USA) to blue formazan products, according to [61]. Saponin (Sigma-Aldrich, Steinheim, Germany) was used as a positive control for inducing cellular death. The results were expressed as a percentage of the control (untreated cells).

#### 4.2.3. Cytotoxicity Evaluation

##### Cell Culture Maintenance

Cell lines were obtained from the American Type Culture Collection (ATCC) and DSMZ-German Collection of Microorganisms and Cell Cultures (DSMZ) biobanks and cultivated according to the supplier’s information.

Breast adenocarcinoma cells (MCF-7; DSMZ: ACC 115), colon adenocarcinoma cells (HTC-15; DSMZ: CCL-225), and prostate cells (DU-145; ATCC: HRB-81) were cultured in RPMI 1640 medium.

Hepatocyte cells (FL83B; ATCC: CLR-2390) were cultured in F-12K medium. Human neuroblastoma (SH-SY5Y; ATCC: CRL-2266) cells were cultivated in DMEM/F12 medium. All media were supplemented with fetal bovine serum (10%) (Biowest, Riverside, MO, USA) and 1% antibiotic/antimycotic solution (Biowest, Nuaill’e, France).

Cells were seeded at an appropriate density to promote optimal growth, and the culture medium was replaced every 48 h. Once cells reached 80–90% confluence, subculture was performed using 0.25% trypsin-EDTA to dissociate the cells, by 2 min and put inside of chamber during 5 min, according to the protocol provided by the biobanks. The cell lines were maintained at 37 °C in a humidified incubator with 5% CO_2_ and 95% humidity.

##### Cytotoxicity Assay

Cells were seeded in 96-well plates (HepG-2: 2.5 ×10^4^ cells; HCT-15: 2.5 × 10^4^ cells/mL; MCF-7: 2.5 × 10^4^ cells/mL; SH-SY5Y: 5.0 × 10^4^ cells/mL; FL83B: 5.0 × 10^4^ cell/mL; DU-145: 5.0 × 10^4^ cell/mL). Cancer cell lines were exposed to compounds (100 μM) for 24 h and vehicle/controls were included to assess potential solvent interference. Cell viability was assessed using MTT assay. Then, the culture medium was removed and 100 μL of MTT (0.5 mg/mL) was added to each cell. Cells were incubated for 1 h in the dark under the same culture condition (37 °C, 5% CO_2_, 95% humidity). After incubation, the intracellular formazan crystals were solubilized with 100 μL DMSO and, after 24 h, the absorbance was measured at 570 nm using a microplate reader (Synergy H1). Cisplatin, tamoxifen, and 5-fluorouracil were used as anticancer standard controls (24 h). Results were expressed as a percentage of control untreated cells and the half-maximal inhibitory concentration (IC_50_) values were determined using nonlinear regression analysis (GraphPad Prism, 8.1, GraphPad Software, San Diego, CA, USA).

### 4.3. Molecular Docking

For the molecular docking studies, MOE 2020 software (2020 0901) was employed. The enzyme structure, obtained from the PDB Database (4BDS), was prepared using the QuickPrep tool (version 2020). Ligands were initially positioned with the Triangle Matcher algorithm, producing 30 poses, which were scored with the London dG scoring function. The top five poses underwent refinement with an induced fit receptor model and were rescored using the GBVI/WSA dG method. The best pose was assessed by comparing it to the expected logical structure and interactions. To validate the docking protocol’s accuracy, the co-crystallized ligand was redocked into the enzyme binding site and compared with the crystallographic pose. Additionally, experimental results and known binding mechanisms of comparable inhibitors were utilized to verify the docking parameters.

## Figures and Tables

**Table 1:** Significant results of the cholinesterase inhibition assays of the synthesized compounds.

Compound	AChE		BChE	
	Inhibition (%) ^a^	*K*_i_ (μM) (Type of Inhibition)	Inhibition (%) ^a^	*K*_i_ (μM) [*K*_i_′ (μM)] (Type of Inhibition)
**10**	39.14 ± 0.9	n.d.	83.82 ± 1.3	0.89 ± 0.08 [2.96 ± 0.39] (mixed-type)
**11**	34.64 ± 1.4	n.d.	65.23 ± 0.9	2.34 ± 0.20 [10.6 ± 1.4] (mixed-type)
Galantamine	88.19 ± 0.5	0.54 ± 0.01 (competitive)	52.00 ± 0.5	9.37 ± 0.67 (competitive)

^a^ % Inhibition at 50 μM.

**Table 2:** Cytotoxicity of the compounds in 3 human cancer cell lines (HCT-15, MCF-7, and DU-145), human neuroblastoma cell line (SH-SY5Y), and normal murine hepatocytes (FL83B).

Compound	IC_50_ (μM)				
	HCT-15	MCF-7	DU-145	SH-SY5Y	FL83B
*δ-Xylonolactam*					
**3**	>100	>100	>100	>100	n.d.
*5′-Azido nucleosides*					
**7**	72.73	>100	>100	>100	22.74
**8**	>100	>100	>100	>100	n.d.
**9**	>100	>100	>100	>100	n.d.
*5′-Guanidino nucleosides*					
**10**	>100	>100	27.63	>100	73.74
**11**	64.07	43.67	24.48	12.14	24.08
**12**	76.02	>100	>100	>100	>100
**5-Fluorouracil**	155.5	n.d.	n.d.	n.d.	>100
**Cisplatin**	n.d.	n.d.	>100	13.92	25.71
**Tamoxifen**	n.d.	27.19	n.d.	n.d.	19.51

n.d.: not determined.

**Table 3:** Calculated partition coefficient (c log *p*) of the tested compounds and reference drugs.

Compound	c log *p* ^a^
*δ-Xylonolactam*	
**3**	1.33
*5′-Azido nucleosides*	
**7**	2.83
**8**	2.84
**9**	2.18
*5′-Guanidino nucleosides*	
**10**	4.50
**11**	4.51
**12**	2.83
*Reference Drugs*	
**5-Fluorouracil**	−0.58
**Cisplatin**	0.43
**Tamoxifen**	5.93
**Galantamine**	1.39

^a^ Predicted using ALOGPS 2.1. Based on c log *p* values, compounds **3** and **7**–**12** are considered as lipophilic (c log *p* > 1), 5-fluorouracil is considered as hydrophilic (c log *p* < 0), cisplatin is considered moderately lipophilic (0 < c log *p* < 1), and tamoxifen and galantamine are considered as lipophilic (c log *p* > 1).

## Data Availability

Data is contained within the article.

## Supplementary Materials

The following supporting information can be downloaded at: https://www.mdpi.com/article/10.3390/ph18050734/s1, Figure S1: A. ^1^H NMR Spectrum of compound **3** in CDCl_3_; B. ^13^C NMR Spectrum of compound **3** in CDCl_3_. Figure S2: A. ^1^H NMR Spectrum of compound **6** in CDCl_3_; B. ^13^C NMR Spectrum of compound **6** in CDCl_3_. Figure S3: A. ^1^H NMR Spectrum of compound **7** in CDCl_3_; B. ^13^C NMR Spectrum of compound **7** in CDCl_3_. Figure S4: A. ^1^H NMR Spectrum of compound **8** in CDCl_3_; B. ^13^C NMR Spectrum of compound **8** in CDCl_3_. Figure S5: A. ^1^H NMR Spectrum of compound **10** in CDCl_3_; B. ^13^C NMR Spectrum of compound **10** in CDCl_3_. Figure S6: A. ^1^H NMR Spectrum of compound **11**; B. ^13^C NMR Spectrum of compound **11**. Figure S7: A. ^1^H NMR Spectrum of compound **12**; B. ^13^C NMR Spectrum of compound **12**. Figure S8: Neuroprotective effects of compounds **8–11** (0.01–10 μM) on differentiated SH-SY5Y cells when exposed to 6-OHDA (100 µM) for 24 h.
