# Supplementary material for: 5′-Guanidino Xylofuranosyl Nucleosides as Novel Types of 5′-Functionalized Nucleosides with Biological Potential
Source: Pharmaceuticals (Basel). 2025 May 16;18(5):734. doi: 10.3390/ph18050734 (PMC12114750; doi:10.3390/ph18050734)
Supplement: Supplementary file 1 [file pharmaceuticals-18-00734-s001.zip › pharmaceuticals-3525096-supplementary.pdf]

Supplementary Materials

5'-Guanidino Xylofuranosyl Nucleosides as Novel Types of 5'-Functionalized Nucleosides with Biological Potential

Jennifer Szilagyi,[1] Tânia Moreira,[1] Rafael Santana Nunes,[1,2] Joana Silva,[3] Celso Alves,[3] Alice Martins,[3] Rebeca Alvariño,[4] Niels V. Heise,[5] René Csuk,[5] and Nuno M. Xavier [1,6]*

[1] Centro de Química Estrutural - Institute of Molecular Sciences, Faculdade de Ciências, Universidade de Lisboa, Ed. C8, 5° Piso, Campo Grande, 1749-016 Lisboa, Portugal

[2] BioISI-Instituto de Biosistemas e Ciências Integrativas, Faculdade de Ciências, Universidade de Lisboa

[3] MARE-Marine and Environmental Sciences Centre & ARNET-Aquatic Research Network Associated laboratory, ESTM, Polytechnic University of Leiria, Av. Porto de Pesca, Edifício Cetemares, 2520-630 Peniche, Portugal

[4] Departamento de Fisiología, Facultade de Veterinaria, IDIS, Universidade de Santiago de Compostela, 27002 Lugo, Spain

[5] Bereich Organische Chemie, Martin-Luther-Universität Halle-Wittenberg, Kurt-Mothes-Str. 2, D-06120 Halle (Saale), Germany

[6] Research Institute for Medicines (iMed.ULisboa), Faculty of Pharmacy, Universidade de Lisboa, Av. Prof. Gama Pinto, 1649-003 Lisboa, Portugal

*Correspondence: nmrmxavier@ff.ulisboa.pt

Table of Contents

1. ^{1}H NMR and ^{13}C NMR Spectra for Compounds **3, 6-8, 10-12** **S1 – S7**

2. Neuroprotective effects of compounds **8**, **10**, and **11** **S8**

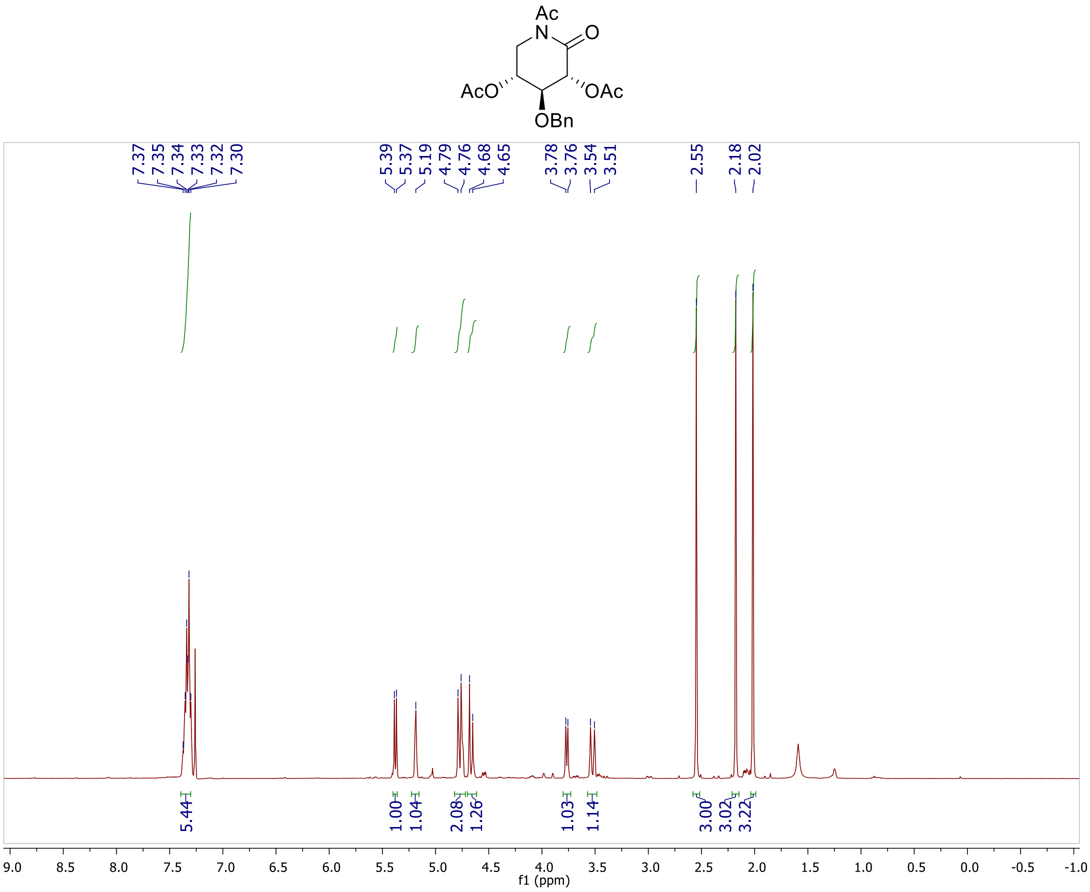

Figure S1 A. ^{1}H NMR Spectrum of compound **3** in $CDCl_3$.[1]

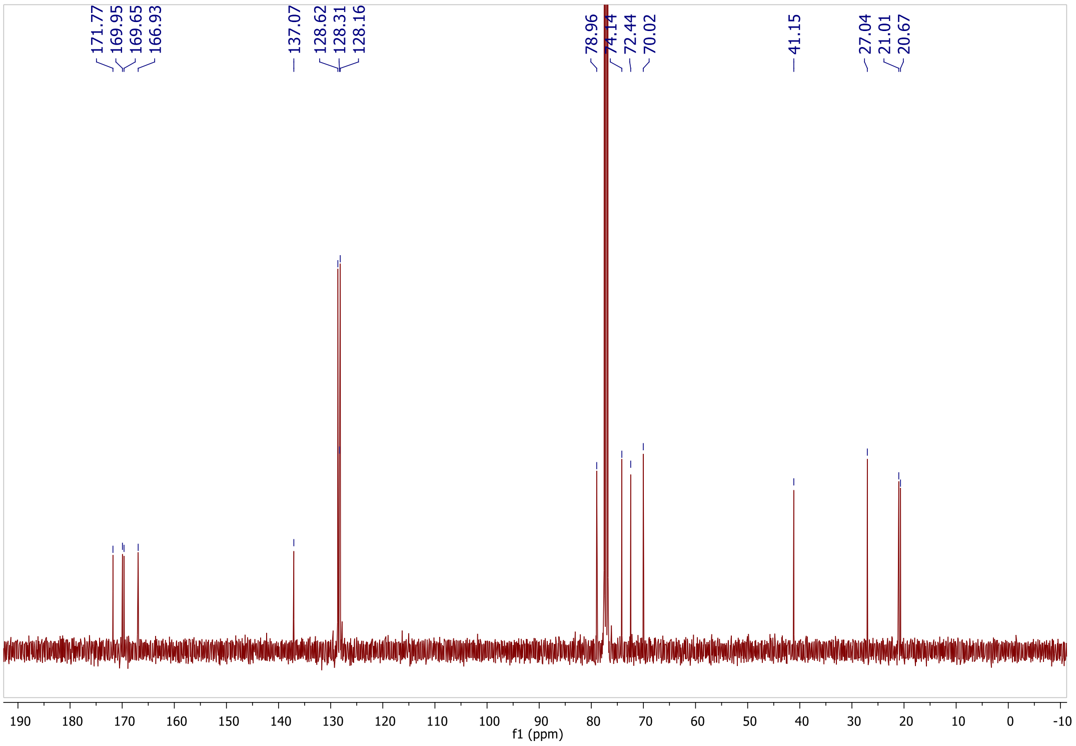

Figure S1 B. ^{13}C NMR Spectrum of compound **3** in $CDCl_3$.

[1] Signal at 1.6 ppm is due to water traces.

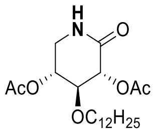

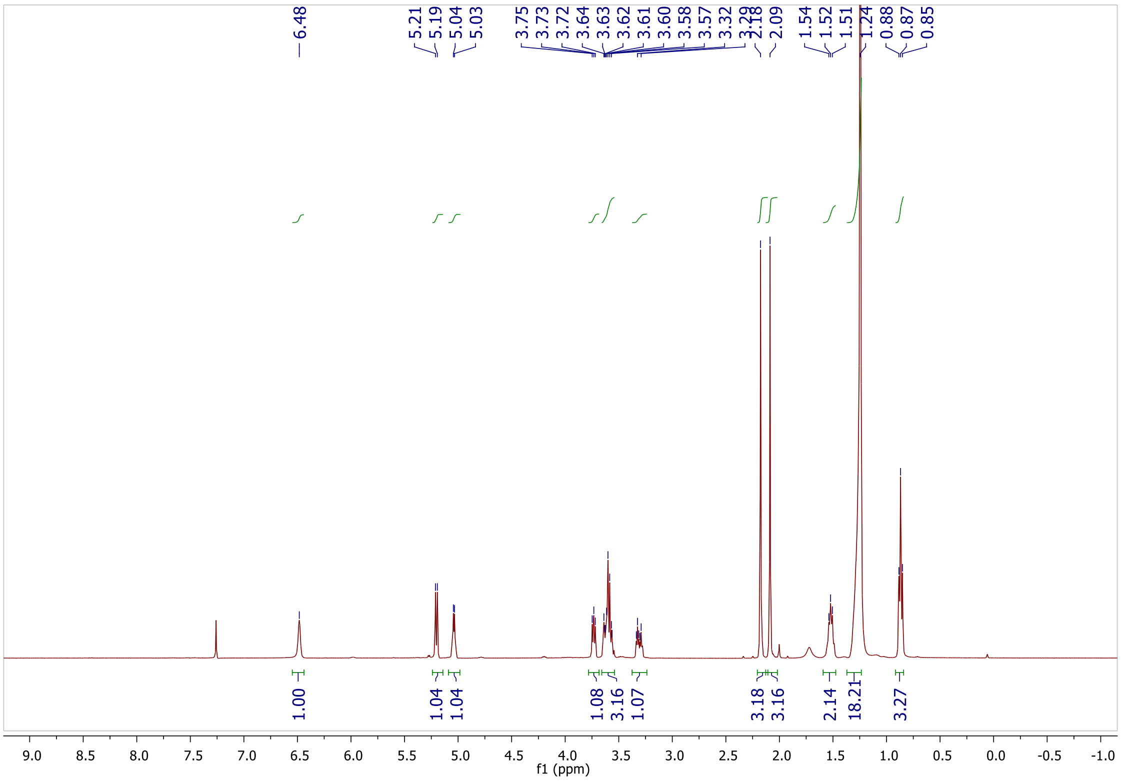

Figure S2 A. ^{1}H NMR Spectrum of compound **6** in $CDCl_3$.

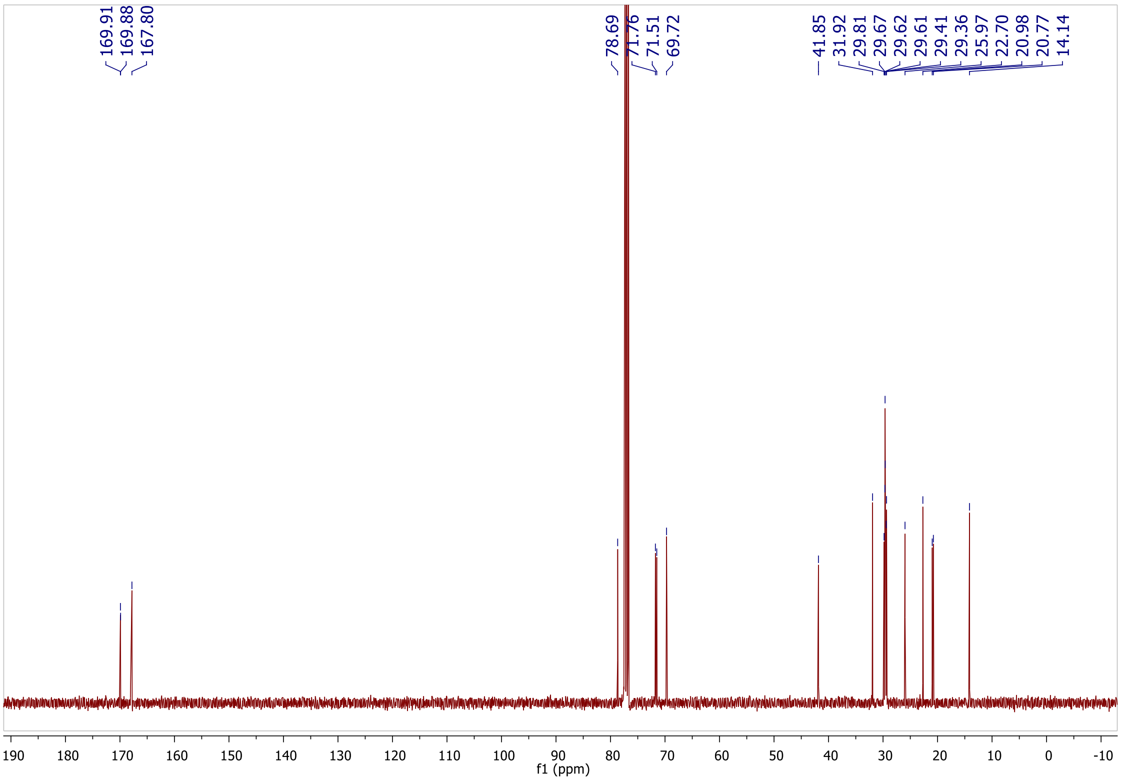

Figure S2 B. ^{13}C NMR Spectrum of compound **6** in $CDCl_3$.

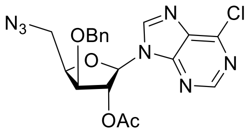

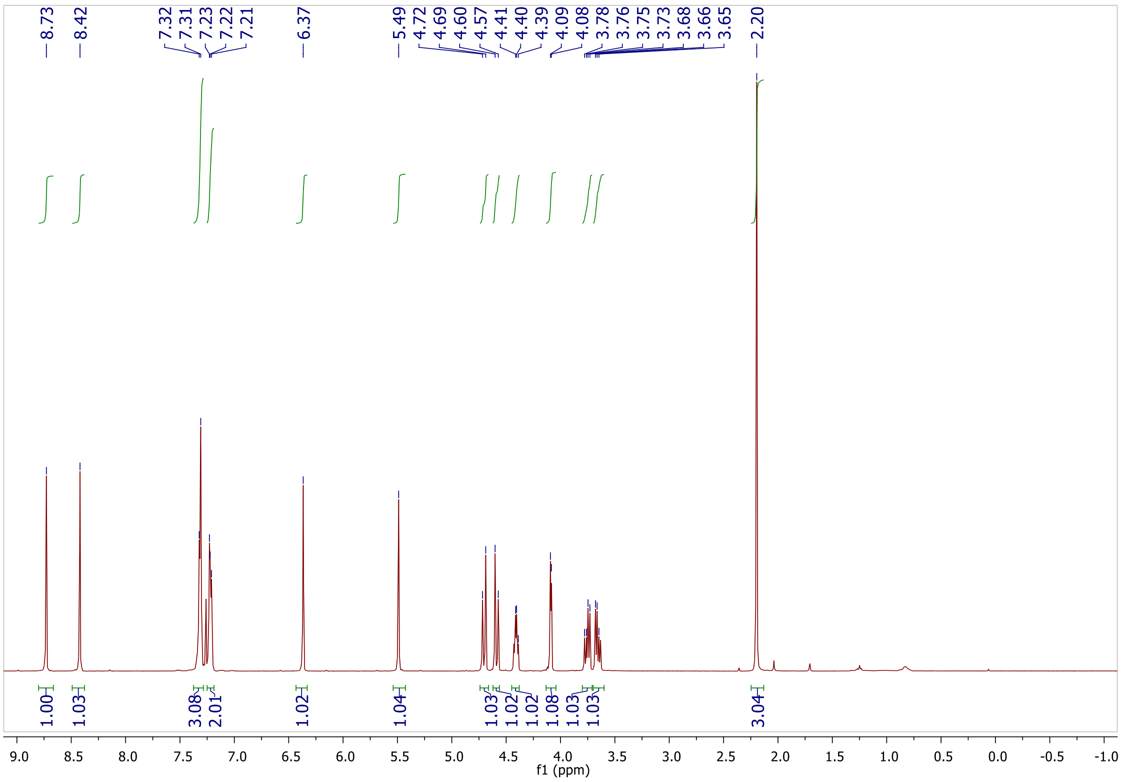

Figure S3 A. ^{1}H NMR Spectrum of compound **7** in $CDCl_3$.

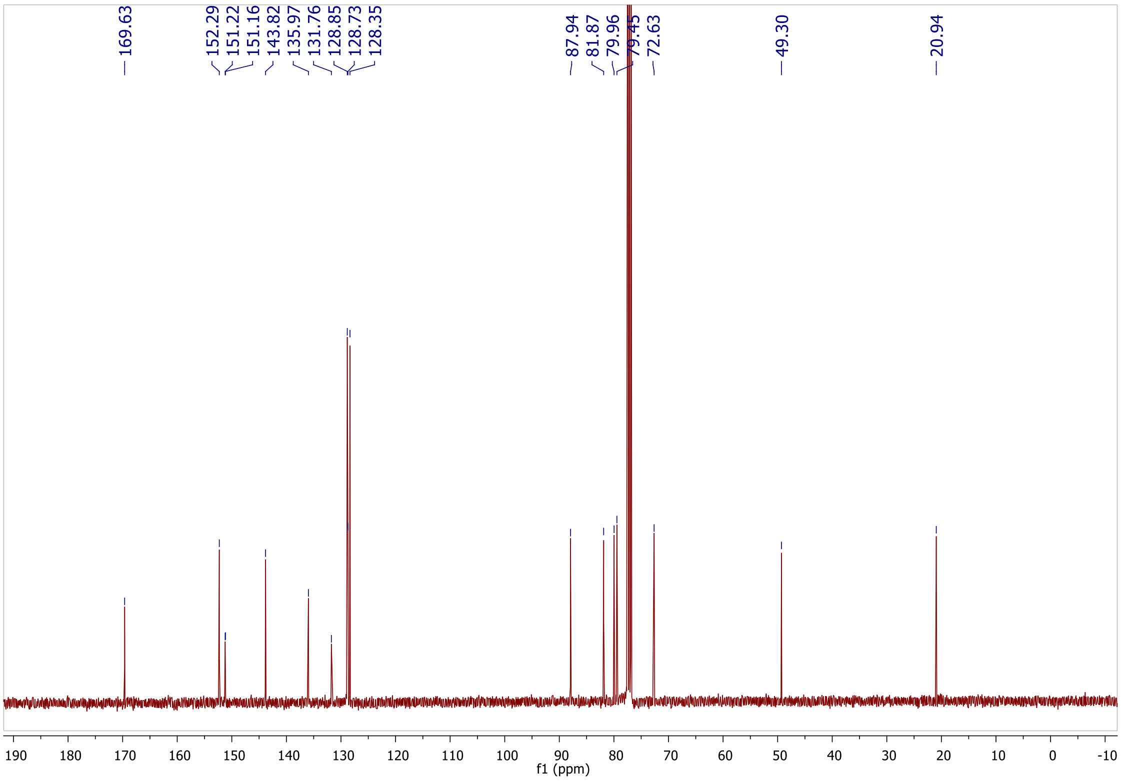

Figure S3 B. ^{13}C NMR Spectrum of compound **7** in $CDCl_3$.

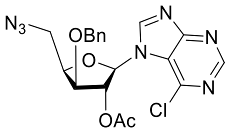

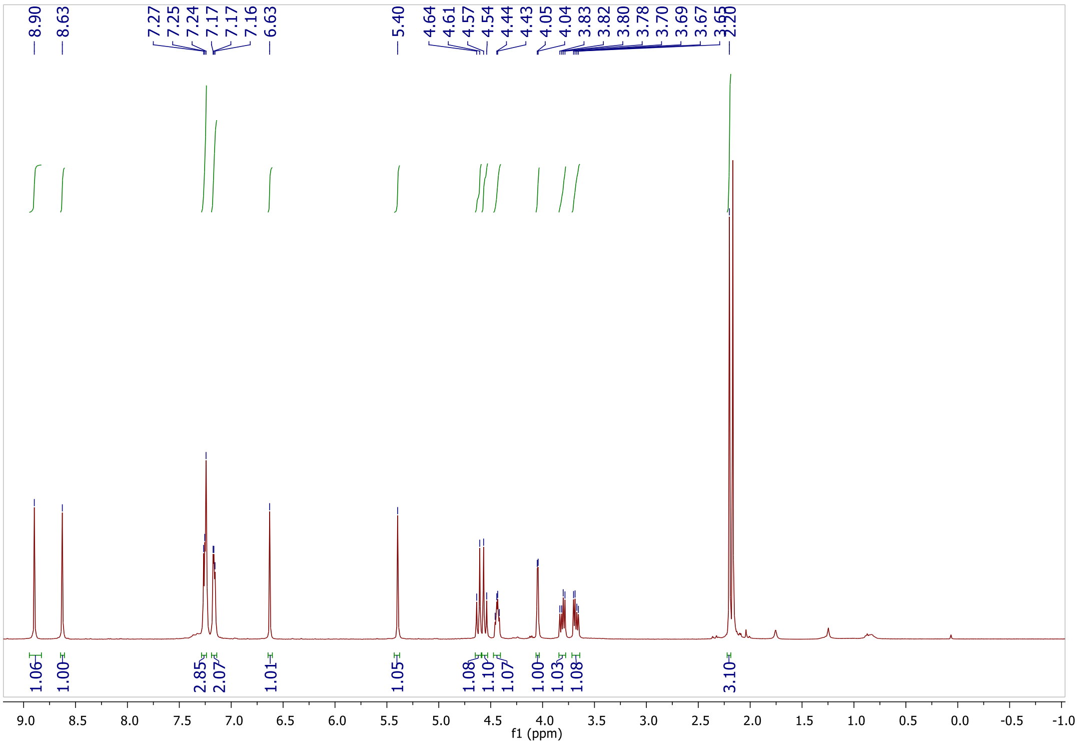

Figure S4 A. ^{1}H NMR Spectrum of compound **8** in $CDCl_3$.[2]

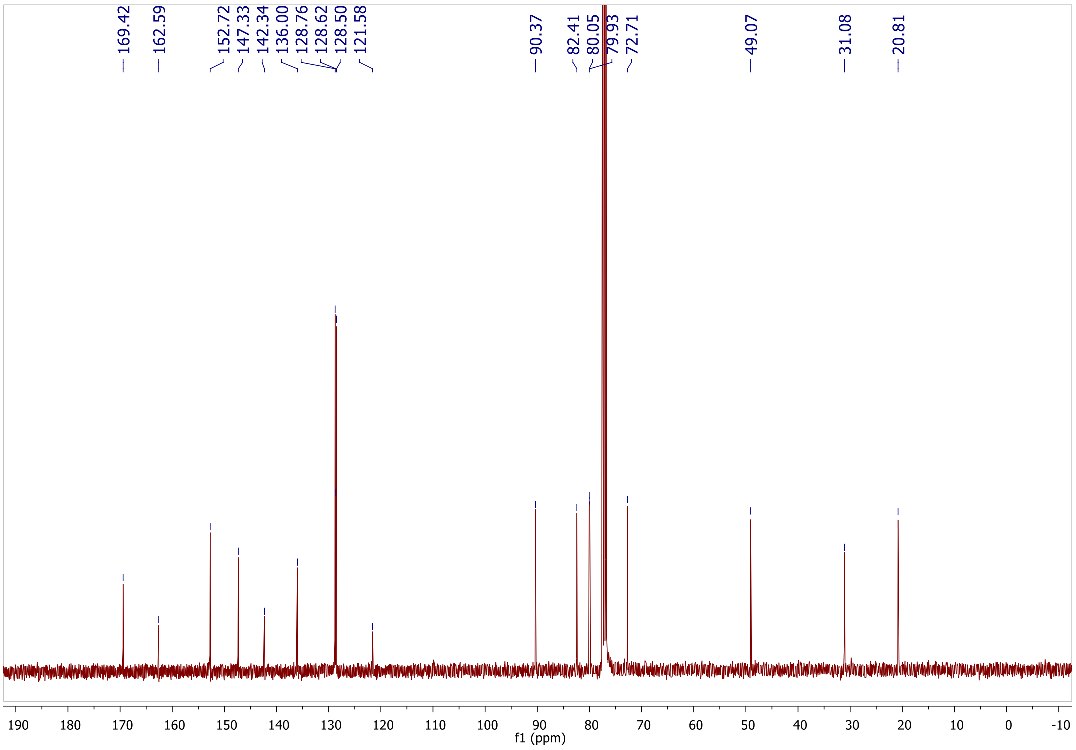

Figure S4 B. ^{13}C NMR Spectrum of compound **8** in $CDCl_3$.

[2] Signal at 2.17 (s) is due to solvent traces (acetone)

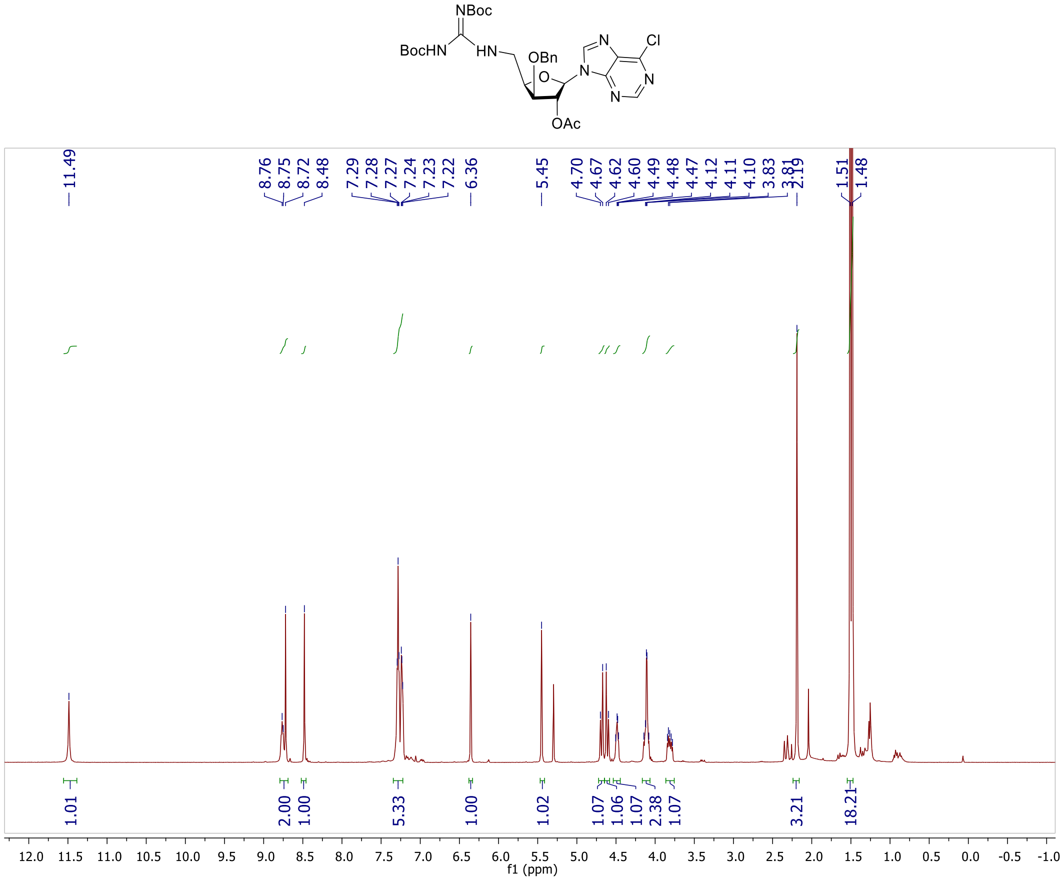

Figure S5 A. ^{1}H NMR Spectrum of compound **10** in $CDCl_3$.[3]

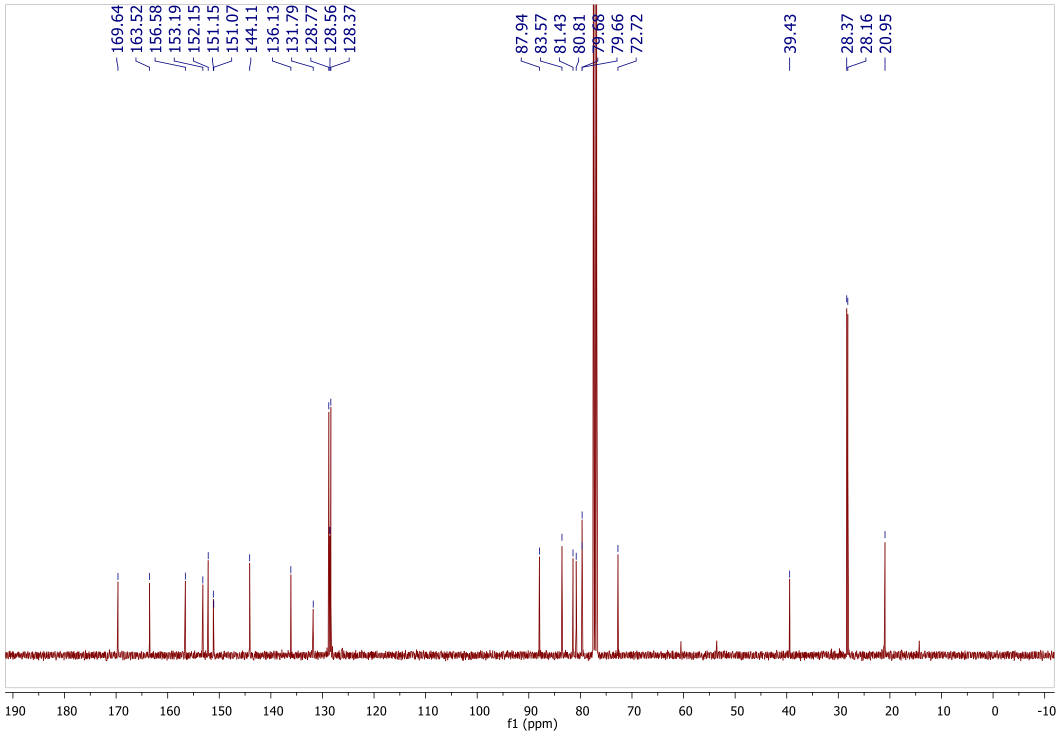

Figure S5 B. ^{13}C NMR Spectrum of compound **10** in $CDCl_3$**.**[4]

[3] Signals at 1.25 ppm (t), 2.05 ppm (s), 5.03 ppm (s) are due to solvent traces (EtOAc and CH_2Cl_2).
[4] Peaks at 14.2 ppm, 21.1 ppm, 53.5 ppm, 60.4 ppm are due to solvent traces (EtOAc and CH_2Cl_2).

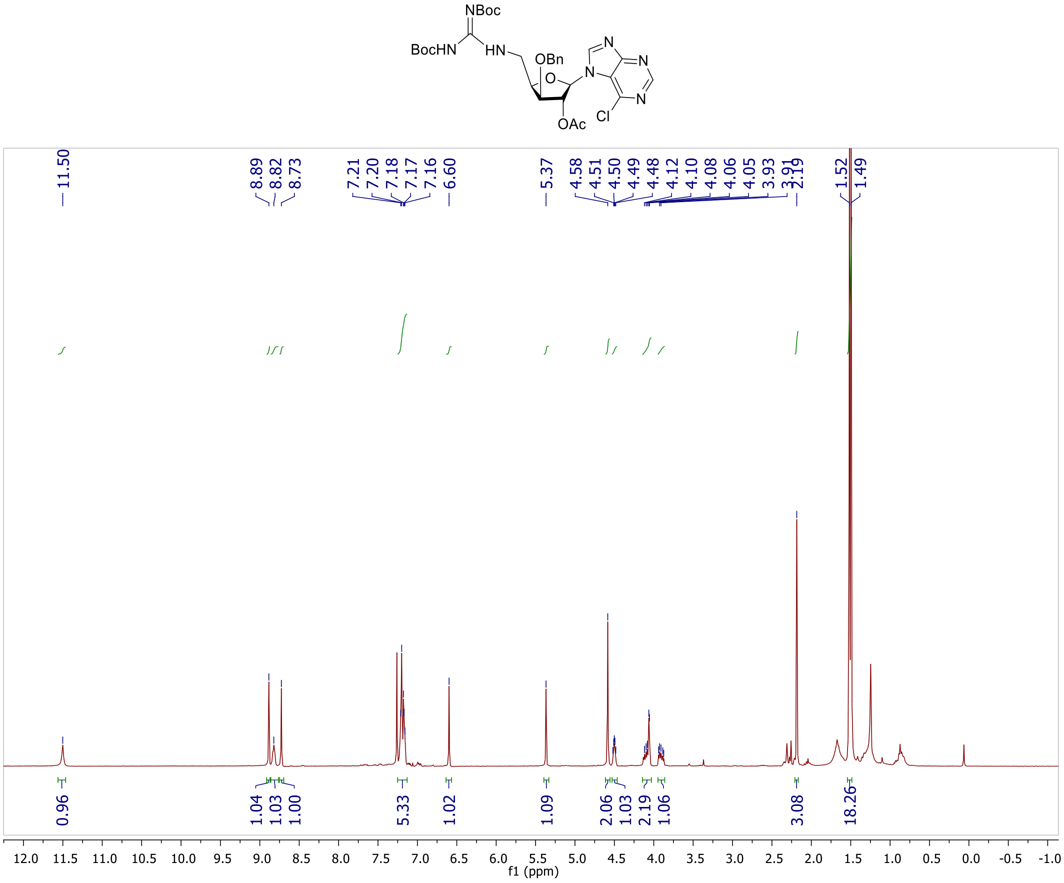

Figure S6 A. ^{1}H NMR Spectrum of compound **11**.[5]

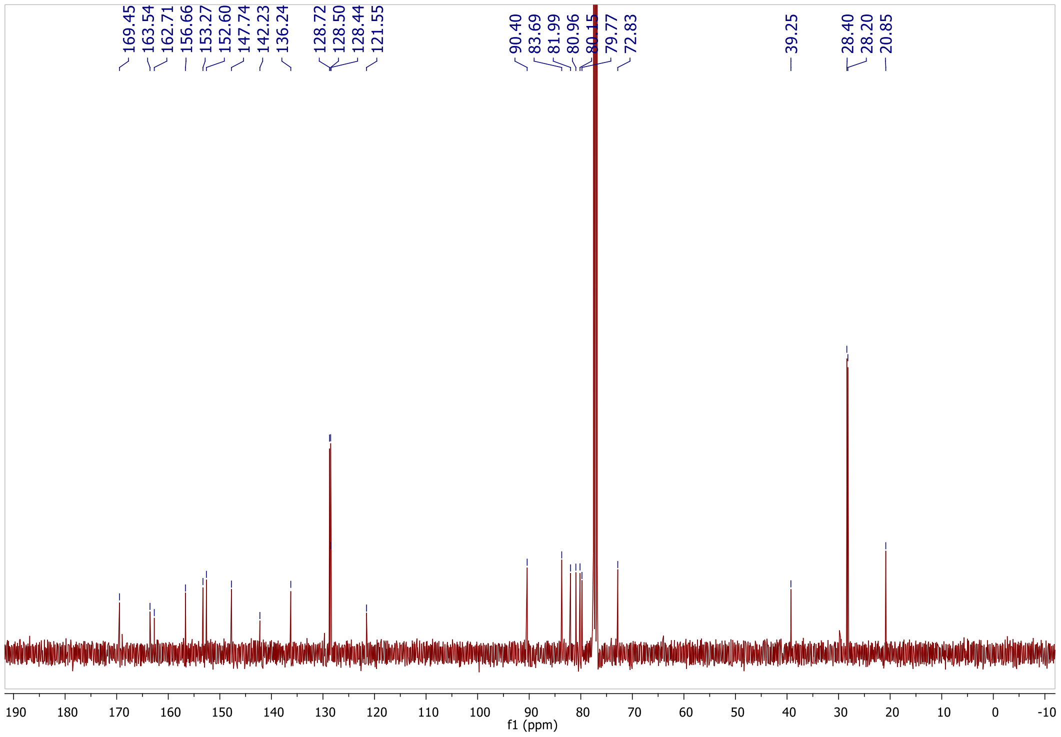

Figure S6 B. ^{13}C NMR Spectrum of compound **11**.

[5] Signals at 1.25 ppm and at 1.6 ppm are due to "grease" and to water, respectively.

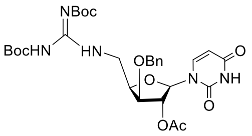

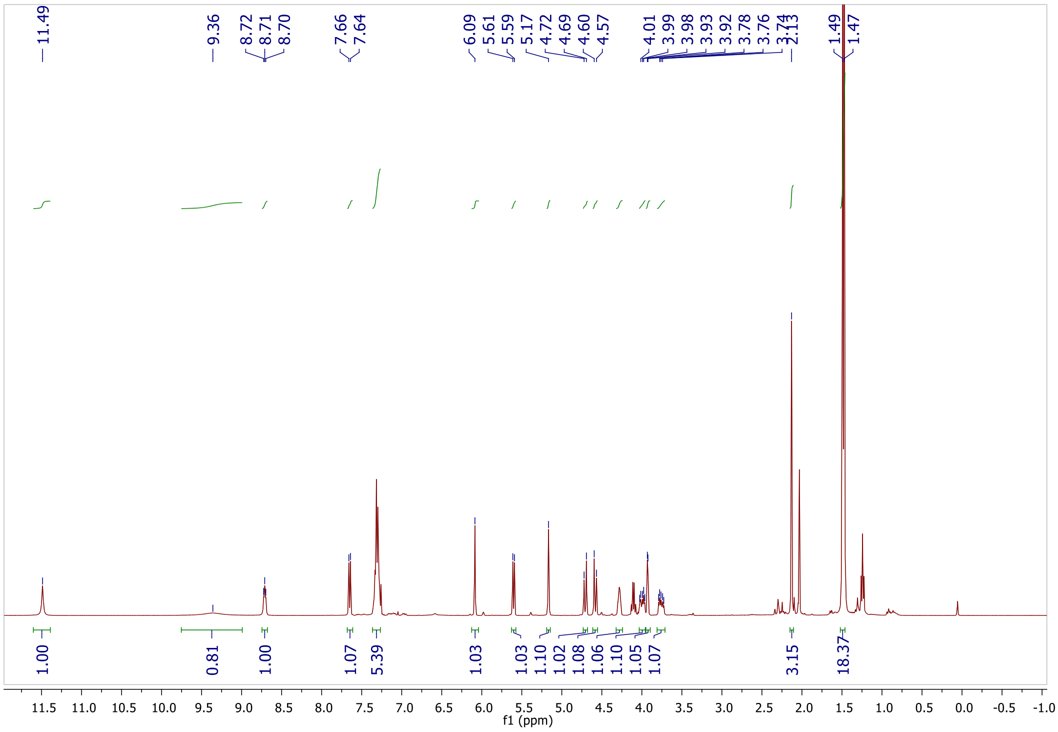

Figure S7 A. ^{1}H NMR Spectrum of compound **12**.[6]

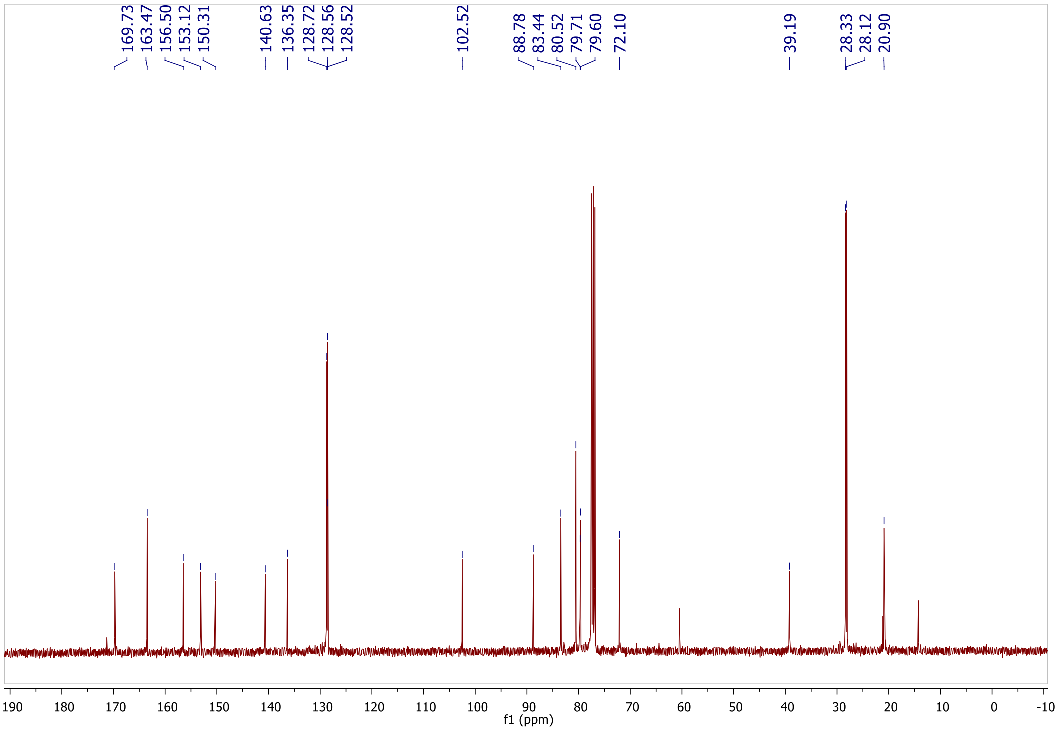

Figure S7 B. ^{13}C NMR Spectrum of compound **12**. [7]

---

[6] Signals at 1.25 ppm (t), 2.05 ppm (s) and 4.12 ppm (q) are due to solvent traces (EtOAc).

[7] Peaks at 14.2 ppm, 21.1 ppm, 60.4 ppm and 171.4 ppm are due to solvent traces (EtOAc).

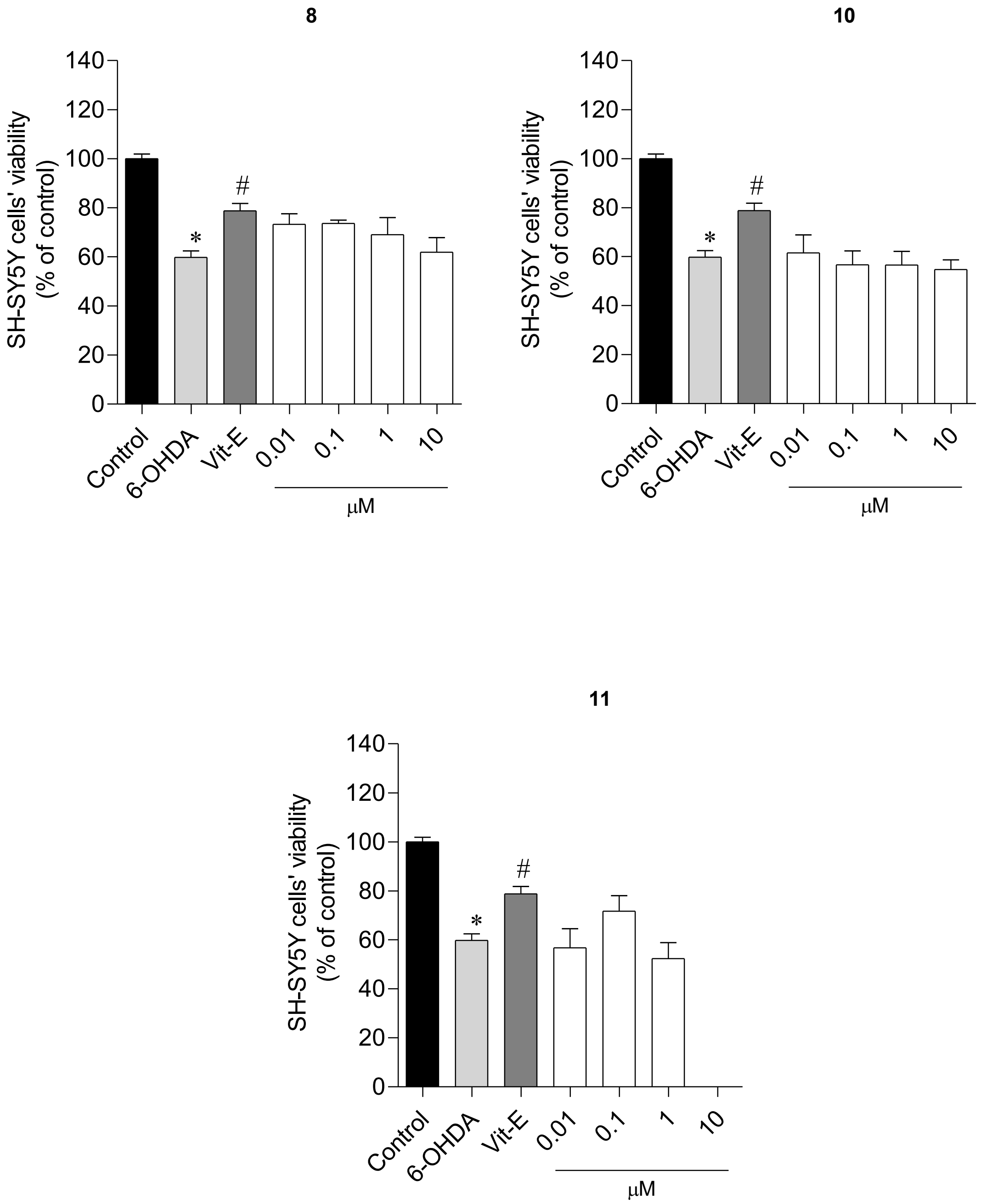

Figure S8. Neuroprotective effects of compounds **8–11** (0.01 – 10 μM) on differentiated SH-SY5Y cells when exposed to 6-OHDA (100 μM) for 24 h.
